# Plant counting: origins, development, and future paths

**DOI:** 10.1016/j.plaphe.2026.100277

**Published:** 2026-09-08

**Authors:** Hao Lu, Wenjing Zhang, Meng Yang

**Affiliations:** National Key Laboratory of Multispectral Information Intelligent Processing Technology, School of Artificial Intelligence and Automation, Huazhong University of Science and Technology, Wuhan, 430074, China

**Keywords:** Plant counting, Plant phenotyping, Computer vision, Benchmark dataset

## Abstract

Plant counting functions as a central role in the quantification system of plant phenotyping. Over the years, the field has evolved through successive technological paradigms, and its modern form has been strongly influenced by advances in visual counting from computer vision. In this review, we synthesize the historical origins of plant counting, assess its current fragmented landscape, and outline a roadmap toward standardized, universal plant counting systems. We propose a coherent conceptual framework— the *four-level hierarchy of plant counting*, which characterizes the environment, platform, sensor, and counting entity in biological organization —grounded in the need of high-throughput plant phenotyping. This framework aims to guide the development of plant counting systems that are not only accurate on individual dataset, but also reusable, comparable, and trustworthy across modern plant phenotyping scenarios. We argue that existing plant counting approaches are constrained by species-specific designs, sensor-dependent assumptions, and local, region-bound assessments. We hope this review can serve as a reference for building cross-species, cross-modal, and cross-scale visual plant counting systems.

## 1 Introduction

High-throughput plant phenotyping (HTP) has become a cornerstone for connecting breeding and cultivation, improving crop yield and plasticity, and optimizing resource-use efficiency under increased climatic variability. Conceptually, plant phenotyping can be viewed as plant-centered health checks: it translates observable signals into quantitative traits that support diagnosis, decision-making, and intervention [1]. The analogy in Fig. 1 highlights three interlinked phenotypic dimensions—morphology, physiology, and biochemical components. Physiological and biochemical traits provide functional and mechanistic insights, whereas morphological traits often serve as a practical entry point for large-scale measurement; these dimensions are complementary and frequently linked through shared quantitative primitives. In this review, the analogy is used as a methodological bridge. It frames counting as a shared quantitative primitive across phenotyping domains. This framing connects plant counting with mature image-analysis and visual-counting ideas from related fields. Representative examples include cell-image analysis tools such as ImageJ and CellProfiler [2,3], as well as density- and regression-based counting paradigms developed in generic visual counting [4,5]. These ideas provide useful methodological references, but they must be adapted to plant-specific constraints discussed below.

**Fig. 1:**
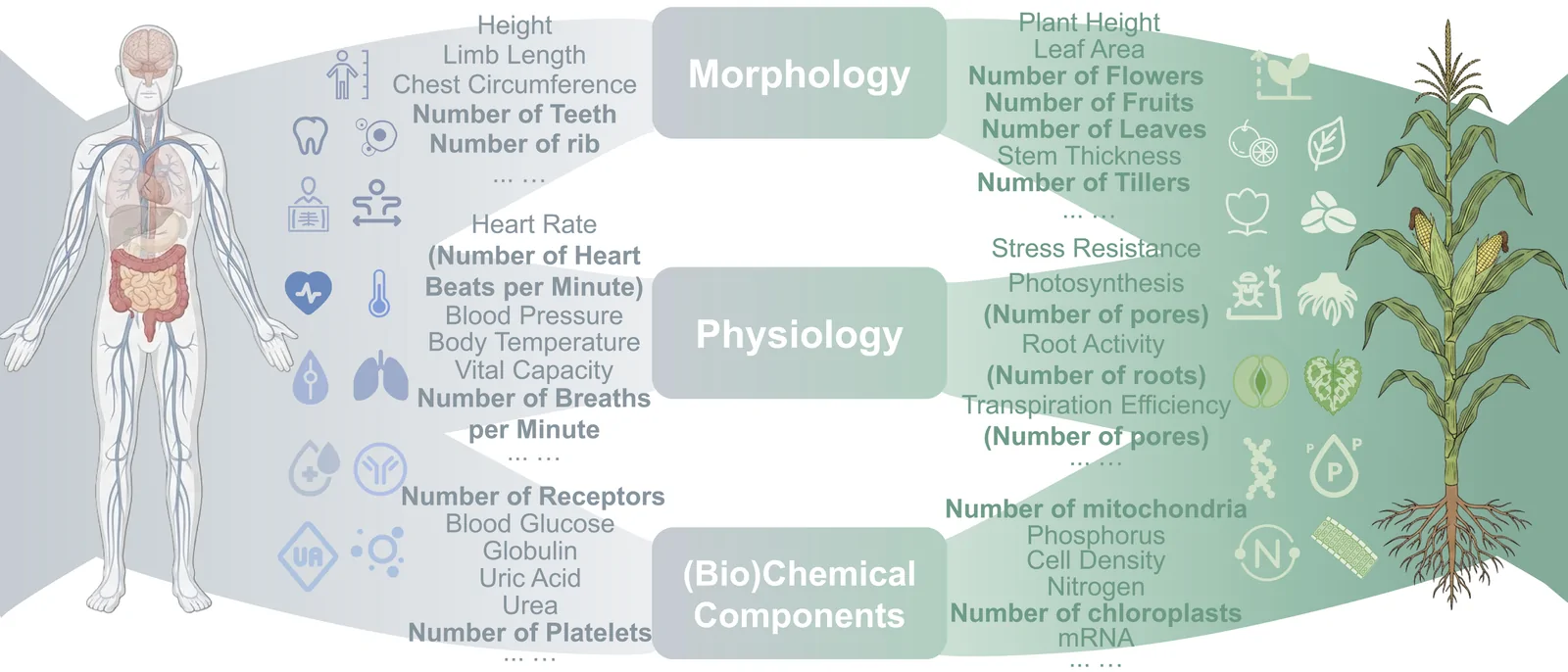
**Counting-related phenotypic parameters are key indicators for both human and plant health checks (phenotyping).** They span across three dimensions including morphology, physiology, and biochemical components, serving as the central role in the quantification system of phenotyping.

Within this phenotyping workflow, plant counting serves as a foundational quantitative primitive that supports broad phenotyping needs across scales and plants [1,6]. In the sense of morphology, countable entities range from whole-plant populations [7] and vegetative organs such as leaves [8] and tillers [9], to reproductive structures such as crop heads/ears [10,11], pods [12], and fruits [13], and further to fine-grained entities such as spikelets [14] or kernels [15,16]. Beyond morphology, counting is also central to physiological and micro-scale assessments, for example stomatal counting [17], phenology-related event counting from time-lapse imagery [18], and cell-level enumeration in microscopy pipelines supported by widely used tools such as ImageJ and CellProfiler [2,3]. Yet, reliable counting at scale has historically relied on manual surveys or laboratory tallies, which are labor-intensive, error-prone, and intrinsically low-throughput [6,19].

To address this scalability bottleneck, computer vision and deep learning have been extensively adopted, typically instantiating one of three paradigms—counting by segmentation, counting by detection, and counting by regression—which parallel the paradigm shift in generic visual counting [4,6,20]. However, unlike generic objects, plants introduce biology-driven constraints, including growth-dependent appearance drifts, order-of-magnitude scale variations (Fig. 2), and non-rigid morphology, which complicates direct transfer and renders reusable solutions non-trivial [21,22]. Despite rapid progress on certain datasets, the current landscape could be summarized by a paradox of *local success and global fragmentation*. Most effort still follows species- and organ-specific pipelines, where models trained within a narrow temporal window or scale often degrade under phenological shifts, seasonal changes, or cross-platform transfers [6,10,22]. The modality landscape is also imbalanced: RGB dominates due to low cost and deployability, while geometric cues (RGB-D/LiDAR), internal-structure modalities (X-ray/CT), and spectral sensing remain under integrated [6]. Even influential public benchmarks remain species-specific, e.g., GWHD for wheat heads [10,23], MinneApple for orchard fruits [24], and ACID for spikelets [14]; each capturing only a narrow slice of the broader space. Furthermore, existing surveys typically organize plant counting either around a single biological object or a single computer vision route [6,19,25], which tends to perpetuate the fragmentation of the field. What remains underdeveloped is a unified hierarchical perspective that aligns the problem definition across plant entities and platforms, a cross-dataset evaluation perspective that supports practical comparability, and an explicit discussion of deployment-oriented generalization as a first-class requirement [10,22,23].

**Fig. 2:**
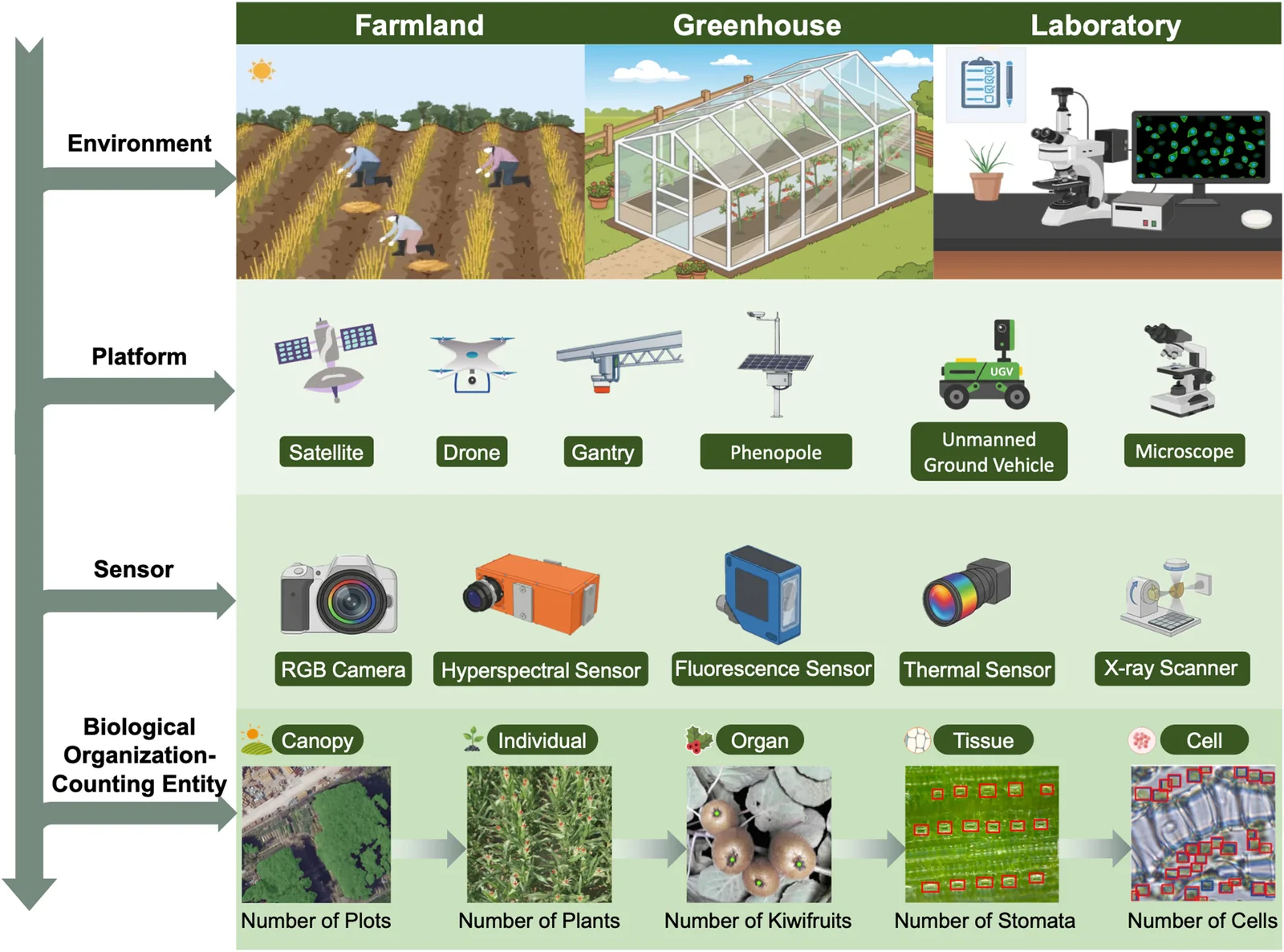
**The four-level hierarchy of plant counting**. There are four interconnected levels that govern data acquisition and define the target scope for plant counting in high-throughput phenotyping: a) *environment*: ranging from outdoor farmland to an indoor controllable laboratory; b) *platform*: using diverse sensing devices, including satellite, drone, gantry systems, and microscopes; c) *sensor*: deploying various imaging modalities such as RGB, hyperspectral, fluorescence, and thermal sensors; d) *biological organization-counting entity*: spanning canopy, individual, organ, tissue, and cell levels, with corresponding counting entities such as plots, plants, kiwifruits, stomata, and cells.

This fragmentation motivates us to rethink the connotation of plant counting for plant phenotyping from a hierarchical perspective, rather than a purely technical one. Such motivation drives us to provide a thorough literature review of plant counting w.r.t. its origins, current status, and future directions. In this review, we define plant counting as *the computational estimation of the cardinality of biologically meaningful plant entities from sensed data (most commonly images)*, potentially accompanied by auxiliary outputs such as localization cues (points or boxes) or dense maps (segmentation masks or density/likelihood maps). We intend to provide insights through moving beyond a catalogue of algorithms to construct a coherent conceptual scaffold. To this end, we introduce the concept of *the four-level hierarchy of plant counting*, visualized in Fig. 2, which depicts environments, platforms, sensors, and biological organization-counting entity levels. It serves as a unified framework to align definitions, datasets, and benchmarks, allowing us to situate and compare diverse counting tasks ranging from microscopic cells to in-field canopies. The four-level hierarchy describes how a typical counting problem is defined step by step. The overarching environment dictates the feasible set of acquisition platforms. The chosen platform, in turn, restricts the deployable sensors. Bounded by its specific optical and physical limits, the selected sensor defines the resolvable biological levels of organization. Finally, this biological level uniquely determines the legitimate counting entities to be quantified. From platform to sensor to counting entity, constraints stack layer upon layer: each stage restricts the next, so the counting problem becomes a highly specific formulation rather than a generic counting template. Counting models are then designed to match that formulation, which naturally produces task-tied pipelines. We note that, current specialized methods may excel under their training regime, but are difficult to generalize when any upstream factor (environment, platform, sensor, or target definition) shifts. It therefore motivates broader research on reusable visual representations, including foundation-style modeling, standardized data resources, and evaluation protocols. To systematically explore these issues, we structure our analysis around three titular questions to construct a coherent narrative. First, to answer where plant counting comes from, we trace the technological lineage from manual surveys to modern vision-based paradigms, elaborating how plant counting has evolved and diverged from generic visual crowd counting [4,20,[26], [27], [28], [29]]. Next, we examine where plant counting is now by operationalizing the proposed four-level hierarchy to reveal the current paradox: while specific models achieve high accuracy on niche datasets, the field remains constrained by species-specificity, modality imbalances, and limited evaluations [6,10,14,23,24]. Finally, to address where plant counting should go, we articulate a forward-looking agenda. We propose a paradigm shift from fragmented, species-specific pipelines toward unified foundation models [[30], [31], [32], [33], [34]], omni-coverage benchmarks [10,33,34], and systematic assessment protocols that ensure reusable, comparable, and trustworthy plant counting systems for next ten-year plant phenotyping [35].

## 2 Where plant counting came from: Technological paradigms and challenges

Here we delve into the origins of plant counting. We commence by tracing the historical progress of plant counting techniques (Section 2.1). Section 2.2 introduces computer vision-based approaches and analyzes how they have progressed along with the advancements in generic visual counting. Finally, Section 2.3 compares plant counting and generic (crowd) counting to elucidate the inherent complexity and unique challenges in plant counting.

### 2.1 A brief history of plant counting: From manual to automated counting

The developmental stages of plant counting technology are briefly summarized in Fig. 3, progressing from manual counting to automated counting. Automated counting methods can be further categorized into sensor-based, image processing-based, machine learning-based, and deep learning-based approaches.

**Fig. 3:**
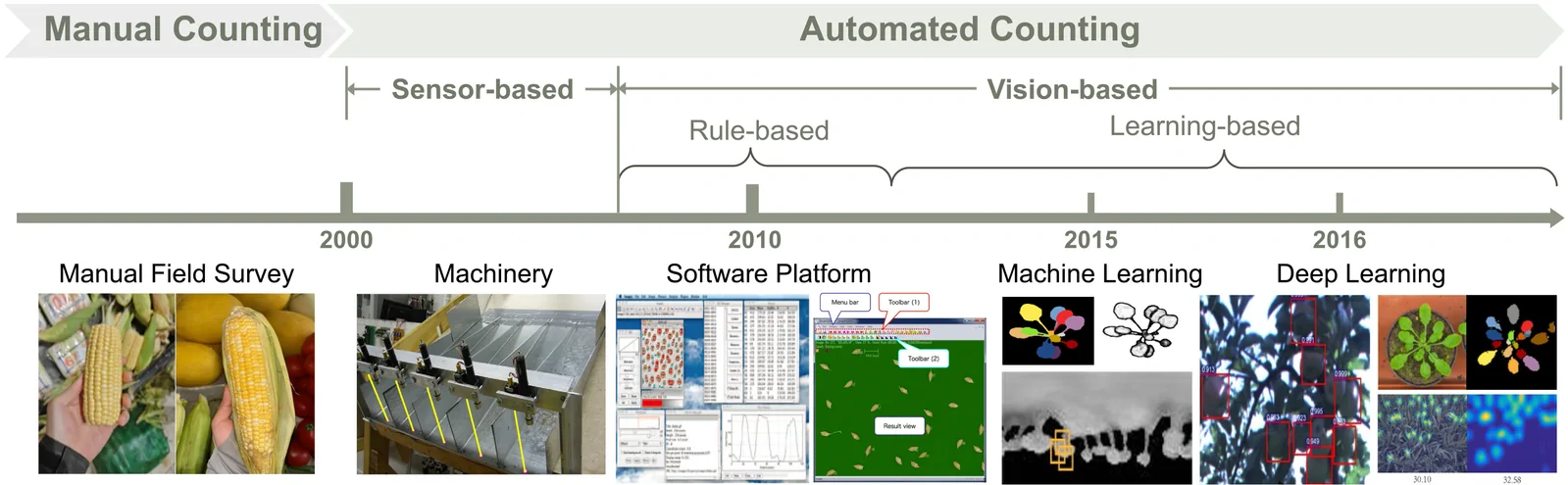
**The evolution of plant counting from manual to automated counting.** The timeline illustrates the shift in plant counting over the past two decades. Manual counting represents the labor-intensive era. The transition to automated counting is marked by two phases: sensor-based methods and vision-based approaches. The latter has evolved from rule-based platforms to learning-based frameworks.

Conventionally, plant counting was accomplished via manual field surveys, where agricultural experts physically counted individual plants or organs and recorded the statistics. However, this process is error-prone, labor-intensive, and low-throughput [6,19]. When immediate estimations of plant counts are required, it is difficult for experts to perform on-site counting within a short period of time. Practitioners began seeking a method capable of meeting high-throughput demands, transforming field data into actionable breeding or management decisions in time.

In the early 21st century, agriculture sectors embarked on the path of automation [36]. As shown in Fig. 3, sensor-based methods were initial trials [[37], [38], [39]]. They operate on converting some physical quantities (e.g., light, pressure, and so on) into some measurable electrical signals, thereby implicitly executing automated counting. Nevertheless, such methods are not only vulnerable to environmental interferences—–such as fluctuating light, temperature, and dust—but also suffer from an inherent deficiency in distinguishing object types, which leads to redundant counting.

To clearly observe and distinguish the counting entities, vision-based methods were introduced and rapidly became the dominant paradigm for plant counting, as shown in Fig. 3. Early methods were image processing-based, such as ImageJ [2], SmartGrain [40], and CellProfiler [3]. They were user-friendly and were designed specifically for plant biology and agricultural science. These software platforms receive digital images of seeds or particles captured by cameras or scanners. The counting value is often output after image calibration, segmentation, separation of adhered targets, and a series of morphological operations. These rule-based approaches are more efficient and automated compared with labor-intensive manual counting or sensor-based methods. However, their performances are often contingent upon uniform lighting and simple backgrounds. When confronted with stochastic outdoor environments, they frequently encounter over- or under-estimations.

To address the robustness challenge, plant counting has later moved to data-driven paradigms. Machine learning, one of the most popular research fields in 2010s, became the first-class choice for agricultural experts [41]. Several plant counting approaches [8,42,43] applying machine learning to agriculture and biology have emerged in workshops such as Computer Vision Problems in Plant Phenotyping (CVPPP) [44]. The purpose of these workshops is not only to identify unresolved challenges, like severe occlusion and variable environments, or to showcase advanced technologies, but also to push the boundary of plant phenotyping. At this time, machine learning has significantly improved the accuracy and robustness of plant counting.

Despite the improved robustness, machine learning-based plant counting also exposed bottlenecks in feature representation and generalization until deep learning is made popular. In particular, deep learning fundamentally reshaped how visual representations were learned and exploited. Early approaches relied on semantic segmentation or object detection to explicitly identify plant instances or organs before counting [[45], [46], [47], [48], [49], [50], [51]]. These methods implicitly assume that accurate instance-level localization is a necessary prerequisite for reliable counting. Most of them applied popular detectors or segmenters to plants, such as ResNet [52], Faster R-CNN [53], YOLO [27], and U-Net [26]. However, when plants exhibit severe occlusion and complex topology, segmentation or detection can probably fail. At this point, TasselNet [21] took a different approach, introducing a local regression strategy to directly estimate maize tassel counts from image patches. This line of work reformulated counting as learning a mapping from visual appearance to numerical counts. Its effectiveness and simplicity showed that, when the final goal is the total count, regression-based formulations can provide a practical alternative to detection- or segmentation-centered pipelines, often with lighter output structures and point-derived supervisory signals. This idea inspired a series of subsequent works that further advanced regression-based plant counting approaches [[54], [55], [56]].

### 2.2 Computer-vision-driven plant counting: On the transition of visual counting paradigms

As revealed in Section 2.1, the evolution of plant counting has been closely intertwined with advances in computer vision. Beyond counting methods, this evolution reflects a deeper transition in counting paradigms. Here, we trace the development of crowd counting in computer vision to delve into how it drives the development of plant counting paradigms. Fig. 4 illustrates the shift in the representation maps of general counting and plant counting since 2000.

**Fig. 4:**
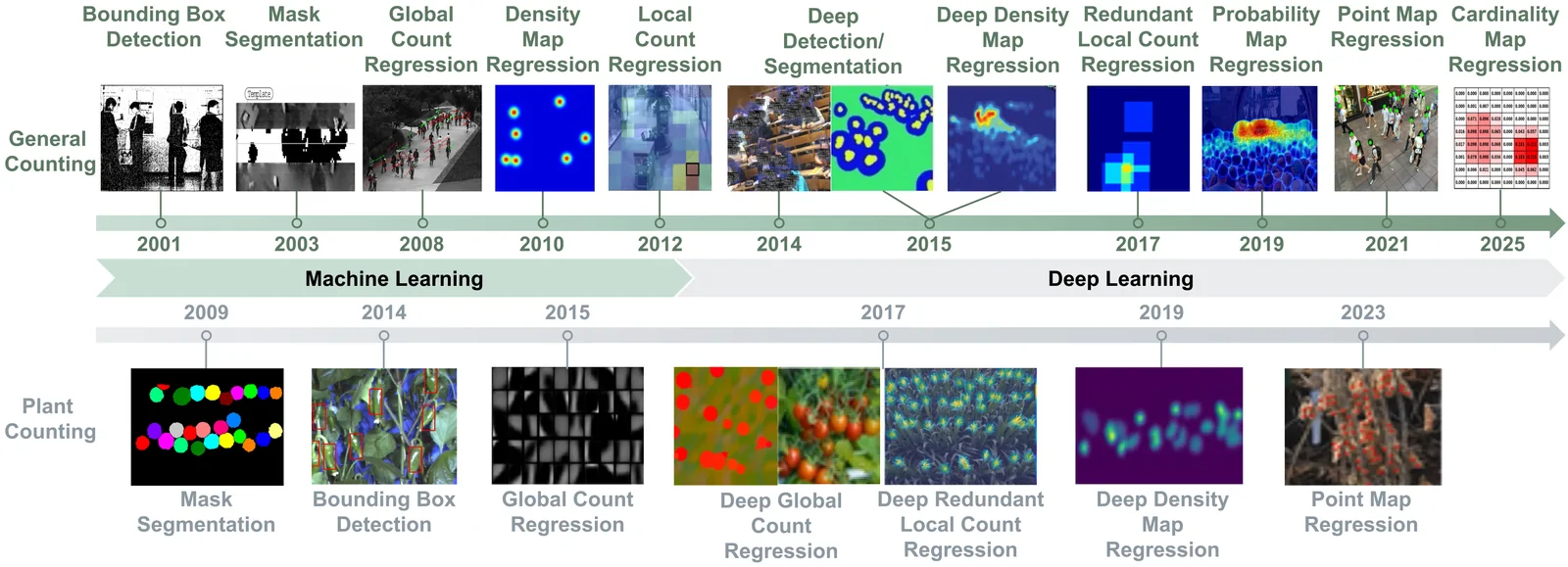
**The evolutionary trajectory of representation maps of counting in the machine learning stage and the deep learning stage.** The upper displays the shift of representation maps of generic visual counting, while the lower shows that of the plant counting.

Early vision-based approaches relied on segmentation masks and bounding boxes as intermediate representations similar to early image processing methods, reflecting their natural roles as explicit cues for counting [[57], [58], [59], [60], [61]]. Representative works segmented the crowd foreground as input before feature extraction and counting [58], extracted local features from segmented blobs [60], or used a box to outline the human head and then computed the number of people [57]. Around 2009, segmentation masks and bounding boxes began to be adopted as intermediate representations for plant counting tasks in agricultural vision systems [42,[62], [63], [64]]. Such intermediate representations could alleviate challenges posed by complex backgrounds, illumination variations, and mildly overlapped plant structures, thereby improving counting accuracy across different plant species and imaging setups.

Inspired by the transductive principle [65], i.e., *one should avoid solving a harder problem than the target application required*, researchers started to rethink the essence of counting, gradually evading localization and segmentation. Initial attempts treated counting as a global regression problem, mapping an entire image to a single scalar value [[66], [67], [68]]. Despite being conceptually simple, such formulations quickly revealed their limitations: they discarded spatial information and failed to model perspective distortion and local density variations. To address these, subsequent studies explored local count regression, estimating counts over image subregions to partially recover spatial cues [69]. However, these methods still lacked a principled way to model fine-grained spatial distributions. A pivotal breakthrough was introduced by Lempitsky and Zisserman [4], which reformulated counting as a density map regression problem. By learning a density function whose integral over the image region equals the corresponding object count, this framework preserved spatial information while avoiding explicit instance localization. Moreover, point-level supervision enhanced the data efficiency of density-based regression compared to per-pixel segmentation. The three regression-based methods above can be summarized as map-based approaches. They employ certain maps as learning targets, forming dense outputs.

The landscape of map-based counting further evolved into redundant count maps [[70], [71], [72]], deep density maps [20], probability maps [28,73], class maps [74], point maps [29], and cardinality maps [75] following the rise of deep learning after 2015 (Fig. 4). Different maps highlight distinct counting challenges. For instance, probability maps [28] were designed for extreme crowd densities, modeling per-pixel object presence likelihood to enable robust counting and localization. To tackle data imbalance, class maps [74] recast counting as a patch-wise classification problem. Critiquing the indirectness and counter-intuition of density estimation, point maps [29] advocated for direct point-to-point prediction. Most recently, cardinality maps [75] address the ambiguity of density values by explicitly encoding object size and shape, offering a scale-invariant representation.

Deep map-based representations are not merely a technical improvement for plant counting; they are a natural fit for the biological and annotation constraints of the task. The adoption of such methods in plant phenotyping began around 2017, closely following advances in general computer vision. Early work by Rahnemoonfar et al. [76] applied a modified Inception-ResNet for global fruit counting, while Lu et al. [21] introduced the first deep local count regression network for maize tassels, later refined in several versions [33,77,78]. Subsequent studies extended density maps to corn kernels [15] and flowering pineapple plants [79]. Inspired by recent point-based paradigms [29], soybean seeds were localized and counted via point maps [80]. This trajectory shows that plant counting has largely evolved by importing and adapting core representation schemes from the broader visual counting community.

### 2.3 Counting plants is beyond counting crowds

Despite the success of deep map-based counting methods, their performances remains fundamentally bounded by the intrinsic and extrinsic challenges posed by the counting entities themselves. A clear understanding of these methods therefore requires examining both the shared characteristics and the critical distinctions between the two domains. Indeed, as shown in Fig. 5, crowd counting and plant counting share common roots and many core challenges such as occlusion, scale variation, and complex backgrounds. They are equally pervasive.

i. *The coexistence of sparse and dense scenes.* For instance, crowd density on the street fluctuates over the course of the day, influenced by factors like crowd movement and the time, e.g., the ShanghaiTech dataset [5]. Plants may exhibit both low and high plant population densities due to natural factors such as soil fertility, as well as agronomic practices including seeding rate and planting spacing, e.g., the Maize Tassel Counting dataset [21]. Typically, sparse and dense scenes lead to a long-tailed data distribution [74]. That is, sparse scenes appear more frequently than dense ones, leading to biased learning in sparse scenarios.
ii. *Complex and diverse background.* When the background and foreground colors are similar, distinguishing the counting entity becomes difficult, which complicates accurate counting.
iii. *Occlusion between instances.* Occlusion encompasses inter-class occlusion between different objects, intra-class occlusion between similar objects, partial occlusion, and complete occlusion. Addressing occlusion poses extremely high demands on model designs.
iv. *Image degradation.* Image degradation may be caused by lens distortion, camera shake during shooting, overexposure due to excessive lighting, or weather conditions such as heavy fog.
v. *Annotation noise.* Manually annotated datasets often exhibit mislabeling, omissions, and inaccurate annotations.

**Fig. 5:**
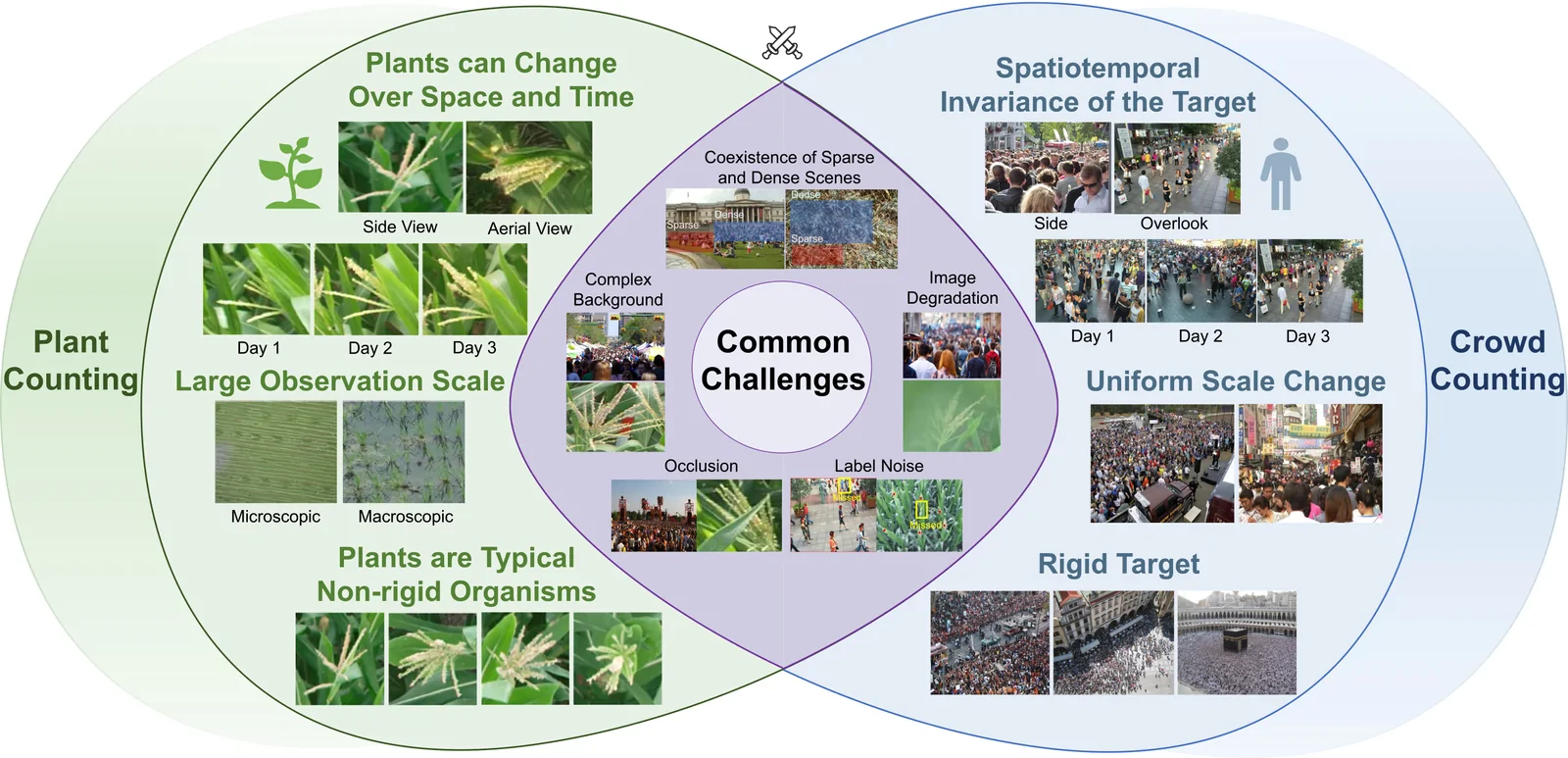
**Common and distinct challenges of crowd counting and plant counting**. While they share certain challenges, domain-specific difficulties of plants appear to be more significant and are shaped by differences in object properties, spatial–temporal dynamics, and observation scales.

However, the unique biological nature and structured growth patterns introduce domain-specific complexities that demand tailored solutions. The key complexities mainly lie in three aspects: dynamics, vast scale variability, and non-rigidity (see Fig. 5).

i. *Plants can change over space and time.* Unlike humans, whose appearance generally remains stable over short observation periods, plants continuously enter new developmental stages in which countable entities may emerge, disappear, or change. For example, flowers may appear abruptly during blooming, fruits may become countable only after reaching a visible size, and senescence or ripening can substantially change the separability of organs from the background. Spatially, plant growth also changes the observation geometry. Taking maize as an example, stalk elongation raises the tassels relative to the camera, causing shifts in viewing angle, apparent scale, and visibility in field images. Together, temporal transitions and spatial shifts in observation make plant counting sensitive not only to appearance variation, but also to changes in the biological existence, visibility, spatial presentation, and definition of the counting entity. Therefore, models trained within a narrow phenological window or acquisition geometry may fail when applied to another growth stage or spatial configuration, even for the same species and organ.
ii. *The scale of plant observation spans orders of magnitude.* As shown in Fig. 2, from microscopic (e.g., cells) to macroscopic (e.g., canopies), virtually any plant structure can be a quantitative target. In contrast, human counting is confined to the macroscopic scale of individuals or torsos, where scale variation arises primarily and predictably from perspective.
iii. *Plants are non-rigid organisms.* Even at a fixed phenological stage, plant organs do not follow a rigid geometric template. Leaves bend and overlap, stems branch, fruits cluster, and organs deform under wind, gravity, or cultivation conditions. This non-rigid morphology makes it difficult to define stable geometric centers or boundaries, thereby complicating the labeling process. In contrast, human bodies have more regular and reusable structural priors, making their morphology easier to model in crowd counting.

As a result, crowd counting methods cannot be directly transferred to plant counting. A clear understanding of these challenges and their implications for the model is therefore essential. Such a systematic analysis forms the foundation for the design of effective plant counting models that explicitly account for the unique characteristics of agricultural scenarios, thereby facilitating their reliable deployment in practical phenotyping applications. Consequently, it is often ineffective to directly transfer crowd-counting methods to plant phenotyping. Several crowd-counting ideas have nevertheless been adapted to plant counting through task-specific redesigns. For dense cereal organs, wheat-head counting methods such as WHCNet [81] inherit the density-map regression spirit of CSRNet, but introduce skip connections and multi-scale feature fusion to better preserve spatial detail under field-scale variation. For discrete soybean pods seeds, point-based formulations such as P2PNet-Yield [82] transfer point supervision and matching ideas to plant counting, while adding plant-specific modules to cope with occlusion, viewpoint change, and yield-related regression. And a more detailed discussion of these adaptations is provided in Section 3. These examples show that the rigid-body assumptions behind human-figure models do not transfer directly to non-rigid plant structures. A methodical understanding of these domain-specific challenges—and their implications for model design—is therefore critical. Such analysis lays the necessary groundwork for developing plant counting models that explicitly encode agricultural priors, ultimately enabling robust and reliable deployment in real-world phenotyping. Analogous to how crowd counting supports surveillance and crowd-flow analysis, plant counting provides a quantitative foundation for downstream agricultural tasks such as yield estimation, phenological staging, and disease monitoring, and thus informs crop management and field decision-making.

## 3 Where plant counting is now: Status quo and datasets

The current landscape of plant counting is characterized by a paradox of “local success but global fragmentation”. While high precision can be achieved for individual tasks, the field faces a systemic challenge of over-specialization. As depicted in Fig. 6, the prevailing workflow is dominated by a “One Species, One Pipeline” paradigm. In this siloed cycle, for every new crop or organ, practitioners must repeat a disjointed cycle: i) collecting data with specific sensors (Fig. 6a), ii) creating task-specific annotations such as boxes or dots (Fig. 6b), and iii) training a model from scratch (Fig. 6c). This framework, despite yielding strong in-domain performance, creates isolated silos and hinders cross-task reusability.

**Fig. 6:**
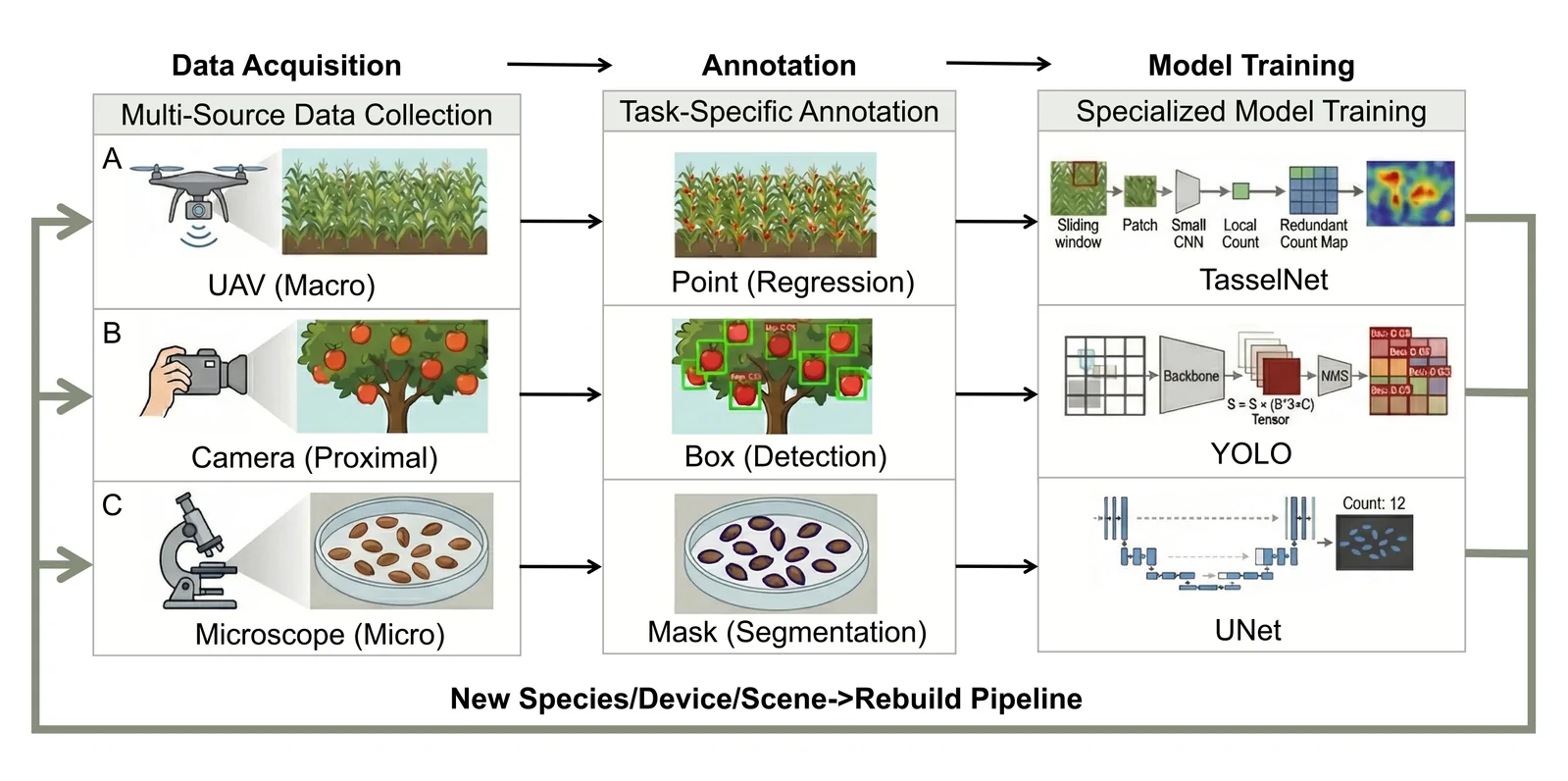
**Current status: one species, one pipeline.** The diagram illustrates the fragmented workflow in plant counting, where changing the target species requires restarting the cycle of data collection, annotation, and model training. (A) Data collection varies by device; (B) Annotation types are task-specific; (C) Models are specialized for single domains.

This section analyzes this phenomenon through four aspects: the siloed nature of species-specific pipelines, the generalization gaps across spatiotemporal scales, the over-reliance on visual modalities, and the fragmentation of data infrastructure. We remark that we do not intend to exhaust all relevant literatures, but to reveal the design patterns behind a bunch of solutions.

### 3.1 Plant counting across species

Current plant counting workflows are inherently organ-specific. Even within a given crop species, different plant organs—such as leaves, fruits, or flowers—can vary substantially in visual appearance, texture, and spatial arrangement, which demands customized modeling approaches. To structure the discussion from an agronomic standpoint, we categorize representative studies into two major groups: cereal crops and horticultural crops. Within each category, we organize existing works following a consistent template: *Target Organ* → *Primary Visual Challenge* → *Methodological Adaptation*. Alongside this agronomic taxonomy, Table 1 provides a comprehensive summary of recent representative methods, categorized by their core computer vision paradigms (segmentation, detection, and regression).

**Table 1:** Representative plant counting methods from 2020 to 2025. The methods are categorized by the counting paradigm and application context.

Paradigm	Year	Species	Platform	Organ	Method	Value	Field	Obs. Scale	Temporal Domain	Ref.
Segmentation	2025	Wheat	RGB	Spike	SDS-Net	Agriculture	Field	Organ	Single	[83]
	2025	Quinoa	RGB	Panicle	Mask R-CNN	Front. Plant Sci.	Field	Organ	Single	[84]
	2024	Lotus	RGB	Seedpod	YOLOv8	Agronomy	Field	Field	Single	[85]
	2023	Wheat	Handheld RGB	Grain	CBAM-HRNet	Plant Methods	Field	Organ	Single	[86]
	2022	Rosette	Top-down RGB	Leaf	Two-stream CNN	Front. Plant Sci.	Lab	Plant	Single	[87]
	2020	Wheat	Handheld RGB	Spike	SpikeSegNet	Plant Methods	Field	Plot	Single	[51]
	2020	Potato	UAV-RGB	Plant	Mask R-CNN	Remote Sensing	Field	Field	Single	[88]

Detection	2026	Sunflower	UAV-RGB	Head	YOLOv11	Sustainability	Field	Region	Single	[89]
	2025	Strawberry	Robot/UGV	Fruit	YOLOv8s	Agriculture	Greenhouse	Plant	Video	[90]
	2025	Barley	UAV-RGB	Head	YOLOv10	Preprint	Field	Plot	Single	[91]
	2025	Soybean	Handheld RGB	Pod	YOLOv8n-POD	Agriculture	Field	Plant	Single	[92]
	2024	Cotton	RGB	Boll	COTTON-YOLO	Appl. Sci.	Field	Field	Single	[93]
	2024	Ginseng	Handheld RGB	Fruit	YOLO-Ginseng	Front. Plant Sci.	Field	Plant	Single	[94]
	2023	Apple	Handheld RGB	Fruit	YOLOv7-tiny	Agronomy	Orchard	Plant	Single	[95]
	2023	Pear	Handheld RGB	Fruit	YOLO-P	Front. Plant Sci.	Orchard	Plant	Single	[96]
	2021	Oil Palm	UAV-RGB	Tree	Faster R-CNN	Agronomy	Plantation	Region	Single	[97]
	2020	Maize	UAV-RGB	Tassel	Faster R-CNN	Remote Sensing	Field	Field	Single	[98]
	2021	Pear	RGB/video	Fruit	YOLOv4+Deep SORT	Sensors	Orchard	Plant	Single	[99]

Regression	2025	Multi-species	UAV-RGB + Handheld	Tassel/Spike/Head	TasselNetV4	ISPRS J. Photogramm. Remote Sens	Mixed	Plot + Field	Single	[100]
	2025	Rice	UAV-RGB	Panicle	LKNet	Plant Phenomics	Field	Plot	Single	[101]
	2024	Wheat	UAV-RGB	Ear	CTHNet (Density Map)	Front. Plant Sci.	Field	Plot	Single	[102]
	2024	Cereal Crops	UAV-RGB	Head/Tassel	Few-Shot Density Regression	Plant Phenomics	Field	Plot	Single	[103]
	2024	Soybean	Handheld RGB	Pod	SPCN	Agriculture	Field	Plant	Single	[104]
	2023	Wheat	UGV (Gantry)	Head (Spike)	WheatLFANet	Plant Methods	Field	Plot	Single	[105]
	2023	Maize	Handheld RGB	Stomata	Density Estimation	Plant Methods	Lab	Micro	Single	[17]
	2022	Apple	Handheld RGB	Fruit	Optimized Mask R-CNN	Front. Plant Sci.	Orchard	Plant	Single	[106]
	2021	Multi-species	UAV-RGB	Tassel/Ear	TasselNetV3	IEEE TGRS	Field	Plot	Single	[56]
	2021	Corn (Maize)	Scanner/Lightbox	Kernel	DeepCorn	KBS	Lab	Organ	Single	[107]
	2020	Tomato	RGB	Fruit	Mask R-CNN	Sensors	Greenhouse	Plant	Single	[108]
	2020	Maize	Handheld RGB	Tassel	TasselNetV2+	Front. Plant Sci.	Field	Plot	Single	[78]

#### 3.1.1 Cereal crops

Cereal crops generally present two key agronomic traits: high planting density and extensive canopy closure. At the field scale, these traits often exhibit homogeneous, texture-like patterns where individual plants are difficult to delineate visually, rendering explicit instance localization, such as bounding box detection, inherently unstable. Moreover, shifting the counting entity from larger, structured organs like inflorescences and spikes to smaller, densely packed units such as seeds and kernels, or from whole-plant seedling counts to microscopic traits, introduces fundamentally different visual characteristics, which in turn triggers distinct modeling paradigms.

##### 3.1.1.1 Dense spikes and interference

For cereal crops such as wheat, rice, sorghum, and barley, spikes are often small in size and frequently occluded by flag leaves. In dense field settings, detection-based methods that rely on bounding boxes suffer from missed detections and over-suppression due to Non-Maximum Suppression (NMS). To circumvent these limitations, many studies have turned to point-supervised or density-map regression frameworks. Representative examples include point-matching approaches such as P2PNet [109] and density/probability-based regressors like CSRNet [28]. Moreover, multi-environment datasets such as GWHD [23] and WED [110] underscore the strong dependence of spike counting on growth stage and field conditions [10,23,110]. Recent architectural innovations have incorporated multi-scale feature aggregation to better detect “tiny and distant” spikes in wide-field imagery, exemplified by WheatLFANet [50]. To further mitigate complex background interference at the field scale, newly designed networks have been introduced, including LKNet [101] for point-based rice panicle counting and CTHNet [102] that fuses local and global cues for high-density wheat ear counting.

##### 3.1.1.2 Extreme density and texture-like phenotypes

Certain plant organs—such as maize tassels, closely packed maize kernels, and rapeseed flowers at peak anthesis—appear in densities ranging from thousands to millions, forming quasi-homogeneous, texture-like canopies that defy conventional instance segmentation. In these “unsegmentable” scenarios, the TasselNet series [21,56,77,78] established a distinct paradigm: direct local-count regression from sub-image patches, avoiding explicit detection or segmentation. Parallel efforts, such as DeepCorn [15], employ dilated convolutions for density-map estimation, enabling accurate counting of densely stacked seeds or flowers by modeling spatial density rather than discrete instances.

##### 3.1.1.3 Geometric irregularities

Economic crops such as soybean pods and cotton bolls often exhibit extreme aspect ratios and random orientations that misalign with the square priors of generic object detectors. A common solution is geometry-aware customization, where techniques like K-Means clustering optimize anchor scales and ratios for specific organ morphologies, significantly improving detection recall [111,112]. To further address these morphological complexities, recent architectural advances have diverged into two distinct pathways. In the detection domain, models such as COTTON-YOLO [93] enhance feature discrimination and bounding box precision for irregular bolls by integrating attention mechanisms and dynamic loss functions. Alternatively, to circumvent severe bounding box overlaps in dense, randomly oriented scenes, approaches like SPCN [104] abandon geometric anchors entirely, shifting instead to a density map-based paradigm.

##### 3.1.1.4 Micro-scale traits

Under microscopic imaging, countable entities such as stomata, cells, and pollen are characterized by extreme small scale, high density and severe texture ambiguity. Phenotyping at this resolution traditionally depends on segmentation or density estimation. While established tools like ImageJ and CellProfiler offer standardized counting workflows for such tasks [2,3], deep learning approaches have significantly improved robustness against complex sample morphology and variable imaging conditions through advanced micro-segmentation or density regression [17,113]. For instance, dedicated density estimation frameworks have demonstrated high efficacy in high-throughput stomata counting [17]. Furthermore, deep segmentation and detection architectures have been actively expanded to enumerate overlapping pollen grains [114] and quantify dense microalgae cells [113].

#### 3.1.2 Horticultural crops

In horticultural crops, countable entities—such as fruits, flower clusters, and individual blooms—are typically discrete, well-separated instances. This structural property makes object detection and instance segmentation natural choices, and facilitates spatially explicit yield assessment. The primary challenges, however, stem from in-field imaging conditions and canopy structures. These include: scale variation due to depth, occlusions that change with viewpoint, adhesion between adjacent organs, and low color contrast in “green-on-green” backgrounds.

##### 3.1.2.1 Perspective-induced scale variance

During ground-based or vehicle-based acquisition, camera-to-canopy distance varies significantly, leading to drastic changes in scale. Detection pipelines have addressed this challenge through architectural adaptations, such as integrating spatial pyramid pooling (SPP) or attention modules into YOLO-based frameworks. These modifications enable adaptive re-weighting of multi-scale features, improving detection consistency across varying depths [24,115]. Although early work relied on global regression to tackle occlusion [76], modern orchard counting increasingly favors detection or segmentation approaches that prioritize scale robustness. Such methods also yield position-aware outputs, enabling per-tree or per-row yield aggregation [116,117].

##### 3.1.2.2 Adhesion and low contrast

Fruits such as grapes, blueberries, and tomatoes often grow in tight clusters, leading to severe adhesion. In contrast, green apples, cucumbers, and immature fruits frequently appear against low-contrast “green-on-green” backgrounds. Since detection struggles to distinguish adjacent instances or to locate low-saliency objects, instance segmentation—typically based on Mask R-CNN and its variants—has become the de facto solution [108]. Some approaches further incorporate depth or multispectral data to improve boundary delineation and reduce false positives [108,118]. For floral targets, which exhibit high shape variability and frequent occlusion, detection-based pipelines enhanced with lightweight backbones or attention mechanisms are commonly used to balance accuracy and efficiency under field-based conditions [119].

Considering both cereal and horticultural crops, a consistent methodological trend emerges: the high density and texture-like appearance of cereal organs favors regression-based approaches, while the structural complexity and geometric variability of horticultural crops drive refinements in detection and instance segmentation frameworks such as anchor design and multi-scale modeling. Yet, as Fig. 7 shows, counting entities in plant phenotyping span multiple biological organizational levels: from landscape-level plots (satellite) and field canopies (UAV) to individual plants and organs, down to microscopic tissues. Existing counting approaches mostly operate at the organ level. This intrinsic multi-scale heterogeneity implies that no single, species-specific pipeline can cover the full spectrum of plant counting tasks.

**Fig. 7:**
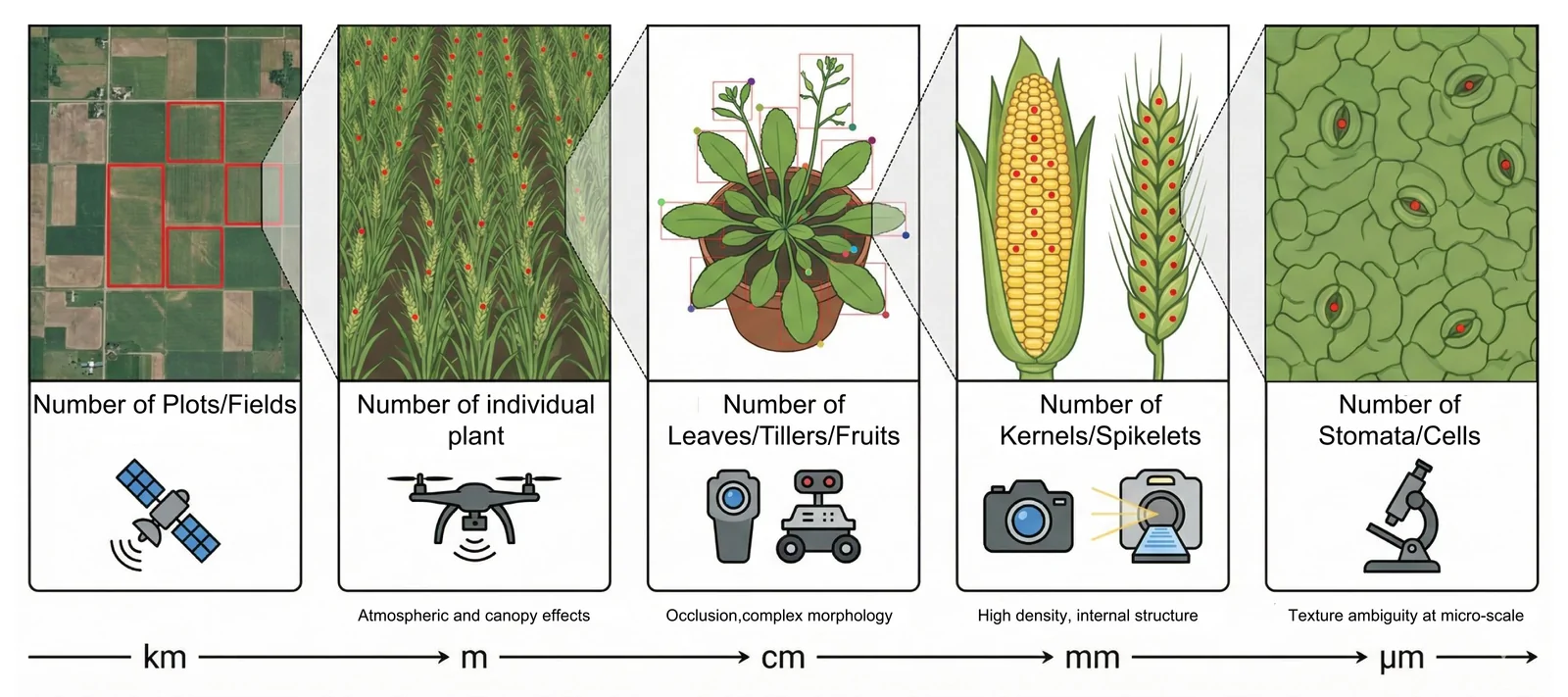
**The multi-granularity task space of plant counting.** The counting entities span across five biological levels of organization, ranging from the regional canopy level (satellite) down to the individual plant, organ, tissue and cell levels. Each level presents distinct challenges regarding the observation scale (km to μm), background complexity, and target morphology.

Taken together, the above applications suggest that the three dominant paradigms of plant counting are not merely alternative implementation choices, but different responses to different structural regimes and application scenarios. In practice, dense cereal organs such as wheat ears, rice panicles, or tassels often favor regression- or density-based formulations, because strong overlap, texture-like canopies, and weak instance boundaries make explicit separation difficult. By contrast, discrete orchard fruits and other relatively isolated targets are more naturally handled by detection-based methods, since their instance extents are clearer and bounding-box supervision is often practical. Microscopic plant structures, such as cells, stomata, or fine tissues, usually require segmentation-based methods, because accurate counting at these scales depends on precise spatial delineation rather than coarse localization alone. Table 2 summarizes these trade-offs in a compact form, highlighting the annotation type, suitable scenarios, and transferability considerations for each paradigm. These differences help explain why no single paradigm currently scales cleanly across the full four-level hierarchy of plant counting. Fig. 7 places these differences within the broader scale- and modality-dependent task space discussed next. Instead, the field needs unified representations and evaluation protocols that can support different biological scales, annotation regimes, and sensing configurations under a common framework.

Remark 1 **(Species diversity and cross-level counting transfer)** For plant counting, a counting entity is not a generic class label: it is tied to plant structure, growth, and agronomic convention. Most datasets fix one counting entity per benchmark, so how counts at different biological levels should compose remains largely open. Research progress requires clear counting semantics. Specifically, it requires rules that define which visual and biological conditions count toward the same counting entity, rather than merely optimizing counting performance for single species–organ combinations. To make such semantics explicit, we suggest characterizing each counting task through five aspects: *(*i*)* the biological level of the target, such as plot, plant, organ, tissue, or cell; *(*ii*)* the inclusion and exclusion rules, such as whether only visible, emerged, attached, mature, or filled structures should be counted; *(*iii*)* the observable evidence and sensing modality required to support the count; *(*iv*)* the annotation granularity and output form, including points, boxes, masks, density maps, or volumetric labels; and *(*v*)* the aggregation rule that links local predictions to agronomic units, such as per-plant, per-row, per-plot, or per-area counts.

**Table 2:** A compact comparison of the three main plant counting paradigms.

Paradigm	Annotation	Scenario	Transferability
Detection	Bounding box	Discrete or moderately separated entities	Struggle in high-density scenes and when morphology changes
Regression	Point	Crowded targets; total count only	Sensitive to representation assumptions and less robust across platforms or resolutions
Segmentation	Mask	Targets requiring precise spatial delineation	High annotation cost and limited transfer across taxa and stages

### 3.2 Plant counting across space and time

Fig. 7 organizes this multi-granularity space and motivates the following analysis of spatial and temporal scalability. Plant counting models are typically developed and validated on imagery from constrained, homogeneous settings. In real-world scenarios, however, counting is often performed under *temporal shifts* (e.g., growth stages, seasons, and years) or *spatial shifts* (e.g., imaging resolutions, fields of view, and sensor altitudes), and even combined spatiotemporal variations (e.g., in video sequences, field robots, or repeated field campaigns). Accordingly, we organize this dimension into three operational regimes: temporal counting (changed time but fixed space), spatial counting (changed space but fixed time), and spatiotemporal counting (both changed space and time, typically along a defined trajectory).

#### 3.2.1 Temporal plant counting

Temporal plant counting evaluates how models behave when observing the same plant population over time. The relevant “time span” encompasses both the physical duration (hours vs. seasons) and the magnitude of phenotypic change across growth stages [120,121].

Most current pipelines are temporally local, operating within narrow windows. For instance, TasselNet [21] focused on the peak pollination (VT) stage, and rosette benchmarks [122] typically cover a 2 ∼4 week window. Under such settings, models face only mild variations; temporal robustness is often achieved implicitly via data augmentation or simple post-hoc smoothing, rather than explicit temporal modeling.

Longer temporal intervals, however, reveal pronounced challenges in model generalization. For instance, in wheat ear counting, models trained on images from the grain-filling stage suffer substantial performance degradation when applied to the ripening and senescence stages (according to the Zadoks scale), primarily due to the marked reduction in visual contrast between ears and the senescing leaf background [11]. Analogously, TasselNetV3 documented inter-annual domain shifts, where regression models failed to transfer effectively across growing seasons because of alterations in canopy morphology [56]. Although sequence-based methods such as ConvLSTM [123] can leverage temporal emergence dynamics to mitigate false positives, the majority of large-scale evaluation benchmarks continue to depend on static train-test splits, which do not explicitly account for seasonal or multi-year domain drift [10,23]. More fundamentally, temporal counting is not only a problem of appearance shift, but also a problem of localization and registration across phenological period. Dedicated plant registration and organ-tracking studies make this limitation explicit: plant correspondences across time must be established under non-rigid growth, changing topology, organ emergence, and architecture-dependent constraints [[124], [125], [126]]. However, this limitation remains largely unresolved in sparsely sampled phenotyping, where the interval between observations is biologically unobserved. For example, a newly emerged tiller or branch may appear close to a previously observed stem or organ, making spatial proximity an unreliable cue for identity. Similarly, a leaf, flower, or fruit may become visible after occlusion, senesce, or shift its relative position between two dates, making it difficult to separate true count change from failed registration. Therefore, future temporal counting studies may need to borrow from trajectory-constrained association, georeferenced revisiting, and architecture-aware 4D tracking or registration; when such constraints are absent, sparse observations should be interpreted as stage-wise cardinality or observed count change rather than guaranteed persistent identities. In summary, static counting models suffer considerable performance loss when encountering growth-stage transitions or inter-annual variation [22]. Consequently, a systematic treatment of temporal shift—for example, through growth-aware model conditioning or explicit adaptation mechanisms—constitutes a notably underdeveloped research direction in automated plant counting.

Remark 2 **(Temporal scalability of counting)** In plant counting, time is not mere domain shift: organ counts can change for biological reasons *(*emergence, abscission*)*, not only because of imaging. At the count level, benchmarks and models should discriminate true count change from appearance change due to occlusion, senescence, or viewpoint. At the instance level, temporal counting should also distinguish persistent identity from stage-wise observation: a target localized at one time point should not be automatically treated as the same countable entity at another time point when biological development makes cross-time registration underdetermined. Together, these count-level and instance-level ambiguities constitute a challenge specific to counting living, growing plants.

#### 3.2.2 Spatial plant counting

Spatial plant counting studies algorithmic robustness under variations in observation scale and coverage. Here the term “spatial span” encompasses both the *physical extent* of observation, ranging from cellular to regional landscape scales, and the corresponding *ground sampling distance*, which can vary from micrometers to hectometers.

At micrometer-to-milimeter scales, objects such as cells, stomata, or seeds occupy tens of pixels. Methods at this scale typically use encoder-decoder segmentation or density regression to delineate closely packed instances within dense micro-structures [17,113]. At milimeter-to-centimeter scales (e.g., organs and individual plants), well-defined shapes and distinct textures provide sufficient cues for standard regression or segmentation pipelines [15,127]. At centimeter-to-meter scales (e.g., plots, rows, and UAV imagery), objects become small relative to the image, backgrounds grow cluttered, and pronounced scale compression occurs. Detectors like Faster R-CNN and YOLO are commonly used, frequently augmented with feature pyramids or tiling strategies to address small objects in large scenes [105,128]. In dense canopies where instance boundaries are ambiguous, density regression is often adopted, typically normalizing scale by processing fixed-size image patches [21,129]. At hectometer scales (e.g., satellite imagery), counting is generally feasible only for large, isolated structures, as finer details are lost.

Currently, scale variation is addressed mainly through manually designed heuristics, such as image tiling and the selection of specific resolutions, rather than through learned, truly scale-normalized representations. Consequently, cross-scale transfer remains a significant challenge: models trained on close-range imagery seldom generalize effectively to UAV views, and vice versa, without requiring extensive re-training or fine-tuning [6,130].

Remark 3 **(Spatial scalability of counting)** For plant counting, changing resolution or platform often changes what *”an* instance” looks like—from discrete objects to texture-like patches—so the same architecture may not even be the right counting formulation across scales. Field deployment needs explicit links between per-tile detections, density maps, and agronomic units *(e.g.,* heads m^−2^*)*, not one-off per-platform retraining.

#### 3.2.3 Spatiotemporal plant counting

Spatiotemporal plant counting leverages continuous observations, typically from videos or repeated scans, to link identities and resolve occlusion. Since consecutive frames overlap, the same instance is observed multiple times such that de-duplication is required to produce more stable counts.

The mainstream methodology follows a *tracking-by-detection* paradigm: applying per-frame detection first and then associating the predictions across time. For instance, Gao et al. [131] combined a lightweight detector with Kalman filtering to count apples from a moving vehicle. Similar pipelines that integrate YOLO-style detectors with general-purpose trackers (e.g., SORT [132], DeepSORT [133], or ByteTrack [134]) have been widely employed for fruit crops to avoid duplicate counting [[135], [136], [137], [138], [139], [140]]. In such approaches, temporal consistency is mainly leveraged for data association, whereas the detector remains frame-based.

Robotic platforms facilitate a tighter coupling between platform motion and plant counting. Zhang et al. [7] employed visual tracking on a field robot to count individual corn plants, while Deng et al. [9] utilized georeferenced maps to revisit and track rice tillers across the growing season. More recently, Feng et al. [141] aggregated soybean counts spatially and temporally using fisheye video sequences. The common mechanism in such systems involves converting per-frame predictions into temporally consistent trajectories that are aligned with the underlying crop row or field structure.

Compared to mobile robots, time-lapse phenotyping systems generate denser temporal data, but they often prioritize tracking over explicit counting. For instance, systems designed to track maize anthesis [18] or Arabidopsis growth [142] produce continuous trajectories rather than discrete counts. Consequently, current models mainly treat the temporal dimension as a source of supplementary frames, rather than a joint signal achieve temporally consistent counting.

Remark 4 **(From local plot counting to spatiotemporal trajectories)** For video- and robot-based plant counting, a dominant failure pattern is often duplicate identities across frames or passes, not a single missing detection. Methods should treat deduplication as part of the counting objective using motion, geometry, and row structure cues, with success judged by stable field-level totals, not frame-wise metrics alone.

### 3.3 Plant counting across modalities

Beyond species and scale, plant counting is also shaped by the imaging modality, as summarized in Fig. 8. Different modalities provide different forms of evidence for defining and separating countable entities. Different sensing modalities capture distinct “slices” of a plant, and thus fundamentally shape what biological entities can be counted and how they should be represented. The progression from RGB and depth sensing to microscopy, tomographic imaging, and spectral imaging alters not only the visual appearance of objects but also the types of feasible supervision and intermediate representations required. For example, these may range from 2D bounding boxes or instance masks to density maps, or volumetric segmentations for internal organs. Therefore, modality selection is essentially a *target-sensor fit* problem: a certain biological trait may necessitate different sensors, while a single sensor can give rise to different counting formulations conditioned on the biological level. Consequently, this heavy reliance on modality-specific designs traps current counting systems in isolated silos. Depending on the sensor and the target, these fragmented outputs are scattered across structurally incompatible data formats: ranging from 2D coordinates or bounding boxes for generic discrete targets, continuous density matrices for crowded scenes, and pixel-wise masks for microscopic cells, to 3D voxel clusters for tomographic internal scans. Since these low-level output structures cannot be directly integrated or algebraically compared, higher-level biological assessments remain disjointed. For instance, a count of external fruits derived from RGB imagery cannot easily be aligned with internal seed counts from CT scans or stress-affected organ counts from spectral data, resulting in isolated and incomparable phenotypic outputs. In what follows, we summarize representative counting pipelines for microscopy, tomographic imaging, and spectral imaging, and discuss why they produce fragmented outputs across different sensing platforms.

**Fig. 8:**
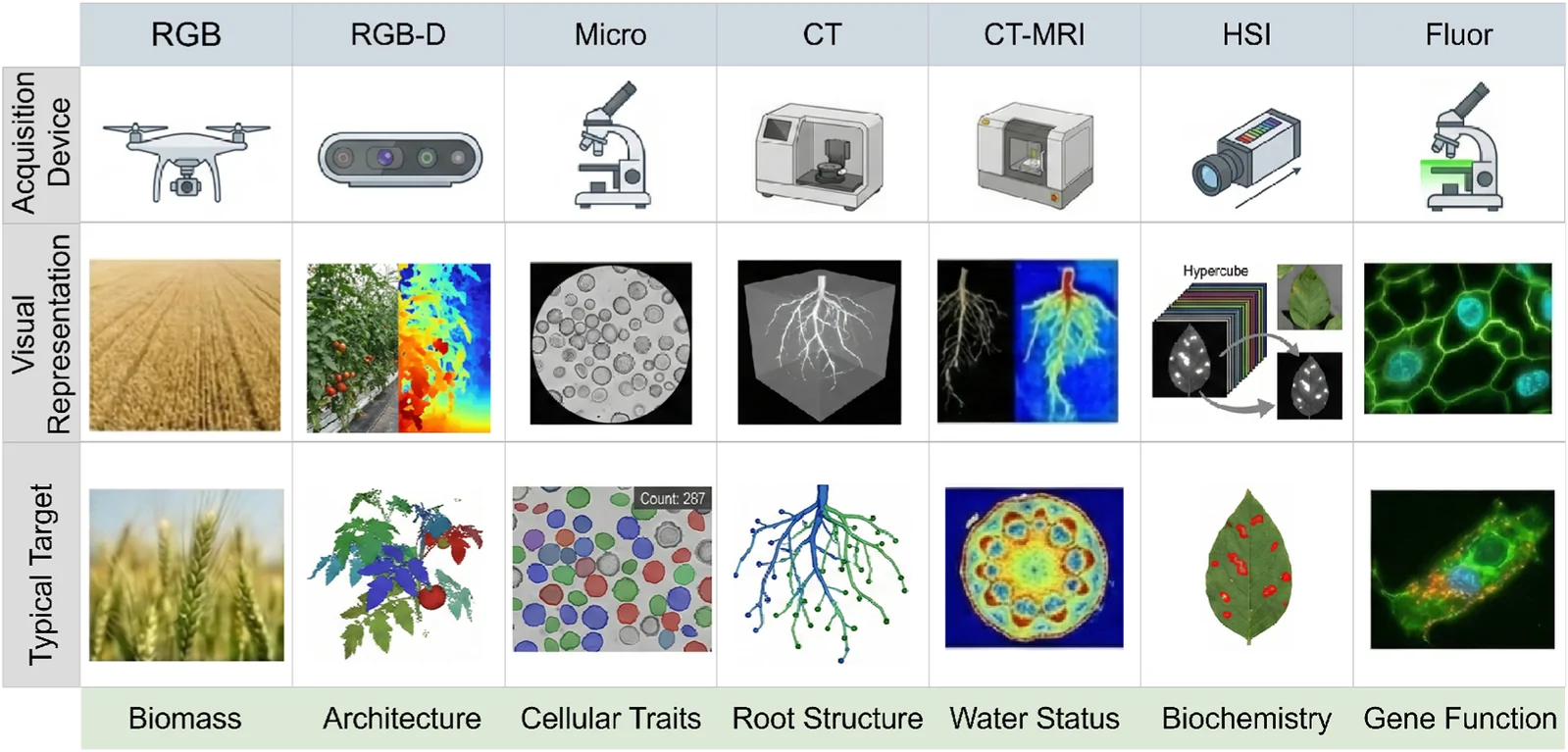
**The spectrum of visual plant counting.** This spectrum matrix aligns seven distinct visual modalities with their corresponding acquisition devices and target counting entities. The classification progresses logically from external morphological traits (captured via RGB or RGB-D) and microscopic features to internal, non-visible structures (imaged by CT or MRI) and physiological states (revealed by spectral or fluorescence data). This illustrates the principle of *target-sensor fit*, demonstrating how dedicated sensing technologies are necessary to resolve biological entities across scales, from canopies down to cells.

#### 3.3.1 RGB imaging

RGB imaging using handheld, gantry, and UAV-mounted cameras is the workhorse modality for plant counting. At the millimetre–to-centimetre ground sampling distances, individual above-ground organs or plants occupy tens of pixels and can be treated as discrete entities. In small-grain cereals, for example, close-range plot images and multi-site datasets such as WED and GWHD provide RGB views at heading, where wheat ears are annotated and counted to approximate yield components, such as the number of ears per unit area [10,23,129]. Similar RGB settings are used for maize tassel counting: Maize Tassel Counting and related datasets capture tassels emerging above the canopy, and local-count regression or detection networks map visible tassels to tassels-per-plot or tassels-per-area [21,56,78]. Rice panicle counting follows the same pattern, with RGB images of panicles near heading serving as the basis for detection- or point-regression-based counting [109,143,144]. In addition, soybean pod and seed counting is performed on RGB images of whole plants or individual ears [112,145,146], while emergence or stand counting for cotton and maize seedlings relies on nadir RGB images [107,111,147].

For orchards and horticultural crops, datasets such as MinneApple and MangoYOLO provide RGB images of apple and mango trees in which individual fruits are annotated; detection networks then convert visible fruits per image into per-tree or per-row fruit counts for yield estimation [24,99,115,148]. These examples collectively demonstrate that a single imaging modality can underlie quantitative analysis at both the organ and the whole-plant level. Across these examples, RGB-based counting primarily informs biomass- and yield-related traits (e.g. heads, fruits, pods, plants per unit area) and coarse architectural properties such as stand establishment and canopy closure.

#### 3.3.2 Depth and 3D sensing

Depth-augmented modalities such as RGB-D cameras, stereo rigs, LiDAR, and multi-view structure-from-motion extend RGB by providing explicit 3D structure in the form of depth maps or point clouds. This additional dimension is valuable when counting entities are heavily occluded or arranged in dense three-dimensional canopies, where 2D projections alone are ambiguous.

One representative pattern is detection followed by 3D association. In orchard systems, 2D fruit detections are often first obtained from individual images and then associated in 3D space to obtain robust final counts. As noted by Bargoti and Underwood [149], fruit detection based solely on 2D imagery in orchard environments is hindered by self-occlusion and overlapping fruits. Expanding on this concept, Santos et al. [150] detected grape bunches in individual RGB images and then associated these detections across multiple views within a reconstructed 3D point cloud. This association ensures that each bunch is counted only once, even if it is partially occluded in some views. Similarly, Ariza-Sentís et al. [151] applied a related approach, leveraging multi-view geometry to resolve overlapping bunches within vine canopies.

Another representative pattern is segmentation or clustering in reconstructed 3D space. In tree-fruit orchards, dense RGB point clouds generated from UAV imagery have been segmented into distinct canopy regions, and fruit detections aggregated in 3D space to estimate fruit loads for individual trees [[152], [153], [154]]. In post-harvest scenarios, RGB-D based instance segmentation enables counting and sizing of overlapping potato tubers in storage crates [155]; the depth information assists in distinguishing individual tubers that appear merged in 2D projections. Across these diverse applications, the integration of 3D information fundamentally shifts the counting paradigm from the question of “how many targets appear in each image?” to “how many distinct physical targets occupy the 3D volume?” This shift directly links quantitative counts to plant architectural features (e.g., tree structure, canopy volume) and volumetric yield traits. Compared with RGB and microscopic images, 3D observations are usually organized as point clouds, voxels, meshes, or reconstructed surfaces. These representations make instance separation, geometric clustering, and cross-view association more direct than image-like density or point-map regression. Accordingly, current 3D plant counting studies remain closely tied to detection, segmentation, and association-based pipelines, whereas regression-based 3D counting is still relatively rare. Broader issues in 3D point-cloud learning and 3D plant phenomics, including reconstruction, detection, segmentation, and generative modeling, have been systematically reviewed elsewhere [156,157].

#### 3.3.3 Microscopic imaging

Microscopic imaging, such as bright-field and fluorescence microscopy, shifts the focus from organs and plants to cellular and sub-organ structures. At micrometer-level resolution, individual cells, stomata, pollen grains, and microscopic seeds can be clearly resolved. Consequently, quantitative counting at this scale serves as a fundamental entry point for quantifying cellular traits and accessing microscopic phenotypes.

Early computational tools, such as SmartGrain and CellProfiler, automated the counting of seeds and cells in controlled microscopic or scanner-based images through rule-based segmentation and morphological operations [3,40]. Deep learning has extended this paradigm to denser and more complex entities. For example, the ACID dataset provides high-res RGB images of wheat ears annotated at the spikelet level. CNN-based models trained on this dataset are capable of localizing and counting individual spikelets along each ear [14], thereby enabling the extraction of fine-grained reproductive traits beyond whole-ear counts. In stomatal phenotyping, U-Net-like encoder–decoder architectures and density regression models have been used to localize and count stomata on leaf epidermal imprints under diverse staining and imaging conditions [17,113]. This approach replaces labor-intensive manual tallying with automated estimates of stomatal density. A similar methodological framework appears in the counting of seeds and grains from high-resolution tray or cob images [107,158], as well as the counting of pollen grains from microscope slides [114]. This framework typically involves high-res microscopic imagery, dense small objects, and deep segmentation or regression architectures that directly convert these visual targets into numerical counts. Collectively, microscopic imaging supports a range of counting tasks that quantify cellular traits, such as stomatal density, cell proliferation, and pollen load, which are directly linked to physiological processes including gas exchange, water-use efficiency, and reproduction.

#### 3.3.4 Tomographic imaging

Tomographic modalities, such as X-ray, Computed Tomography (CT), and micro-CT, produce volumetric image data that reveal internal anatomical structures invisible to surface-based RGB imaging. X-ray CT and micro-CT have been widely reviewed as tools for three-dimensional plant imaging across tissues and organs [159,160]. Their practical use still depends on image contrast, spatial resolution, scan time, and downstream image analysis. While less common in high-throughput plant phenotyping, these modalities are critical in controlled experimental settings where the counting of internal organs or structures constitutes the trait of interest. Thus, tomographic counting depends less on the visibility of targets in a two-dimensional image and more on their separability within a reconstructed volume. In soil-filled columns, CT imaging can reconstruct three-dimensional root architectures, enabling the subsequent counting of root tips, lateral branches, or nodules through 3D segmentation and skeletonization. This strategy underlies CT-based root phenotyping workflows. It supports high-throughput root-system visualization, soil–plant volume segmentation, and repeated-scan analysis of temporal root architecture [[161], [162], [163]]. Similarly, tomographic scans of ears and fruits can resolve individual seeds or kernel compartments within opaque parental tissues, thereby facilitating the direct quantification of filled versus unfilled kernels or internal cavities. Micro-CT also supports reproductive trait quantification when targets are enclosed within opaque tissues. Internal wheat grain compartments and oilseed rape seeds in pods can be extracted from reconstructed volumes and analyzed in three dimensions [164,165]. At the tissue level, X-ray micro-CT can support 3D segmentation of fruit parenchyma cells. Its accuracy, however, depends on sufficient density contrast at cell interfaces [166]. When integrated with MRI-style contrasts, tomographic imaging can additionally map parameters such as water distribution or tissue density. This capability makes it possible to count or quantify functionally distinct internal regions, such as vascular bundles or zones characterized by high versus low water content, within stems or fruits. In all these cases, tomographic imaging fundamentally extends the scope of plant counting from a focus on externally visible features to a probe of concealed internal anatomy. This shift enables the quantitative analysis of root system architecture, internal grain-filling patterns, and structural integrity—metrics that are inaccessible to RGB or depth imaging alone.

#### 3.3.5 Spectral reflectance imaging

A prominent application is crop-weed discrimination, where spectral cues, such as reflectance in the red-edge and near-infrared (NIR) regions, help distinguish weeds from crops in complex backgrounds. Chebrolu et al. [167] integrated multispectral imaging with machine learning to perform pixel-level segmentation of sugar beet plants and weeds in field environments, effectively mapping the spatial distribution and number of weeds within crop rows. Related studies based on field and UAV platforms have similarly demonstrated that the inclusion of NIR or multispectral channels improves the classification and mapping of densely intermingled weeds and crops [[168], [169], [170]]. Large-scale vegetation datasets, such as CropAndWeed and PhenoBench [171,172], extend this idea across species and environments. Models trained on such datasets generate pixel-wise classification maps for multiple plant species and background categories; these maps can subsequently be aggregated to obtain counts of weed patches, crop plants, or vegetation clusters per plot. Spectral reflectance imaging also supports disease and stress detection. Reflectance signatures can reveal symptomatic regions that are challenging to detect reliably using only RGB data [173,174]. In such applications, segmentation outputs can be converted into quantitative counts, such as the number of symptomatic leaves, plants exhibiting stress symptoms, or affected patches within a field.

#### 3.3.6 Fluorescence imaging

Fluorescence imaging measures *emitted* light under controlled excitation (rather than reflected ambient illumination), providing a complementary view of plant function and physiology that is often weakly expressed in RGB appearance [175]. In practice, fluorescence images are commonly interpreted as functional “activity maps” (e.g. photosynthetic responses quantified by chlorophyll fluorescence parameters, or stress-related emissions), where the primary goal is to identify *where* a process is active rather than to recover precise organ geometry [176]. From a counting perspective, fluorescence pipelines typically follow a sequence of steps: fluorescence signal acquisition → background correction and normalization → segmentation of fluorescence-positive regions → connected-component or instance segmentation → the derivation of object counts and spatial statistics. This enables counting (or quantifying) functionally defined targets such as the number of pre-symptomatic infection spots or lesions (fluorescence-abnormal regions) on leaf surfaces [176]. The approach also supports high-throughput disease phenotyping where the thresholding or segmentation of chlorophyll fluorescence images (e.g., maps of the maximum quantum yield of Photosystem II, denoted as *F*_*v*_/*F*_*m*_) is used to quantify diseased area and monitor symptom progression [177]. Beyond stress and pathology studies, fluorescence imaging is widely used in reporter-based screening assays, where fluorescence-positive seedlings or organs can be detected and counted in a high-throughput manner [178]. Compared to reflectance-only spectral imaging, fluorescence can offer stronger contrast for certain functional or physiological states. However, its efficacy often benefits from the geometric context provided by co-registered RGB or 3D sensing data, such as organ boundaries.

#### 3.3.7 Multi-modal fusion

The utility of spectral information is maximized when integrated with structural data from RGB or depth sensors. RGB and 3D sensing provide geometric information, delineating the boundaries and individual instances of plant organs. Concurrently, spectral reflectance and fluorescence data identify the biochemical or physiological classes to which these organs or regions belong, thereby enabling their targeted quantification [179]. Recent progress in plant phenotyping has demonstrated the successful fusion of snapshot spectral imaging with RGB-D data to produce 3D multispectral point clouds. This approach provides a practical framework for aligning high-dimensional spectral signatures with precise 3D structural information at a per-point resolution [180]. Such integration paves the way for multimodal counting systems. In these systems, for instance, counts of heads or fruits can be stratified according to their stress or disease status, directly linking morphological counts with underlying biochemical and functional phenotypes.

Remark 5 **(Counting plants from surface appearance to internal structure)** Plant counting aims at agronomically meaningful counts *(e.g.,* fruits per tree and filled seeds per ear*)*, not modality-agnostic scene parsing. Across modalities, the evidence that defines an instance changes in an ordered way. Surface-structural imaging, including RGB, RGB-D, LiDAR, and multi-view reconstruction, defines instances through visible boundaries or recoverable geometric clusters. Microscopy defines instances through optically resolvable micro-structures, such as cells, stomata, pollen grains, or small seeds. Tomographic imaging defines instances through reconstructed internal structures, and therefore depends on voxel-level contrast, volumetric resolution, and 3D segmentation or skeletonization. Spectral and fluorescence imaging define regions or entities through physiological or biochemical contrast, often requiring co-registered structural masks to provide geometric support. Therefore, counts are not interchangeable unless the biological entity, sensing assumption, and aggregation rule are specified together.The open problem is joint interpretation: what is counted, under which sensor assumptions, and how external versus internal counts relate for the same biological question.

### 3.4 Plant counting fuels: Platforms and datasets

The rapid progress in plant counting over the past decade has been driven by a continuous influx of publicly available datasets collected across diverse platforms. Table 3 summarizes representative benchmark datasets, which span a variety of counting entities, image resolutions, and dataset sizes across different plant species, imaging platforms, and environments. Collectively, these data resources form the foundation upon which the majority of current approaches are trained and evaluated.

**Table 3:** Public datasets for plant counting.

Dataset	Year	Counting Entity	Annotation Type	Image Size	Images Numbers	Reference
Date fruit	2019	Date fruit	Classification label	224 × 224	8079	[181]
SugarBeet2016	2017	Sugar beet, Weed	Semantic mask	1296 × 966	11,552	[182]
CottonWeedDet12	2023	Cotton, Weed	Bounding box	Various	5648	[183]
GWHD	2020	Wheat ear	Bounding box	500 × 500	6615	[10]
ACFR-Multifruit	2016	Apple, Mango	Bounding box	Various	3702	[149]
Bayberry	2019	Bayberry tree	Semantic mask	5472 × 3648	3690	[184]
PRL	2016	Arabidopsis	Segmentation mask	256 × 256	3285	[122]
YOLOPOD	2023	Soybean pod	Bounding box	4752 × 3168	2243	[112]
MrMT	2023	Maize tassel	Bounding box	3648 × 2736	1968	[185]
WSC	2019	Wheat ear	Point	1216 × 912	1764	[77]
MangoYOLO	2019	Mango	Bounding box	500 × 500	1730	[115]
MinneApple	2020	Apple	Instance mask	1280 × 720	1000	[24]
SPIKE	2018	Wheat spike	Bounding box	Various	335	[186]
wGrapeUNIPD	2022	Grape bunch	Bounding box	4288 × 2848	373	[187]
MTC	2017	Maize tassel	Point	3648 × 2736	361	[21]
MTDC	2020	Maize tassel	Bounding box	Various	361	[188]
Fuji-SfM	2020	Apple (2D+3D, SfM)	Instance mask + 3D location	Various	870	[13]
KFuji RGB-DS	2020	Apple (RGB-D-S)	Fruit location	Various	967	[189]
CitDet	2024	Citrus fruit	Bounding box	Various	579	[190]
BlueberryDCM	2024	Blueberry	Bounding box	Various	140	[191]
WEDD	2019	Wheat ear	Bounding box	6000 × 4000	235	[11]
SHC	2018	Sorghum head	Dot/region annotation	1154 × 1731	92	[43]
CVPPP	2016	Leaf (Rosette)	Instance mask	Various	441	[122]
WGISD	2020	Wine grape	Boxes + subset mask	2048 × 1365	300	[118]
ACID	2017	Wheat spikelet	Point	1956 × 1530	500	[14]
URCAL	2024	Rice	Point	1600 × 1600	365	[192]
EWS	2021	Wheat	Semantic mask	350 × 350	190	[193]
PhenoBench	2024	Crop & Weed	Semantic + instance/panoptic mask	1024 × 1024	2179	[172]
CropAndWeed	2023	74 species	Box + semantic mask + stem point	Various	8034	[171]

#### 3.4.1 Platforms

Plant counting platforms span multiple environments and sensing configurations (Fig. 2), ranging from controlled laboratory setups to large-scale field campaigns.

In controlled environments such as laboratories and greenhouses, images are often captured by scanners, light boxes, top-down cameras, or microscopes. This results in high-resolution imagery featured by relatively clean backgrounds and stable illumination. Such controlled settings enable fine-grained counting of small plant structures, such as seeds, spikelets, stomata, and render dense forms of supervision, including instance masks or point annotations, more feasible.

In contrast, field-based phenotyping now routinely uses handheld or fixed pole-mounted cameras, gantry systems, ground robots, and UAVs as standard outdoor platforms. These platforms operate under conditions of variable illumination, wind-induced motion, and cluttered backgrounds. Consequently, they typically face severe occlusion and greater appearance variability. To maintain stable counts, they may require strategies such as image tiling, tracking-by-detection pipelines, or domain-robust feature representations. For UAV imagery, aerial acquisition expands spatial coverage while reducing the perceived size of individual targets. In such imagery, objects can become small and texture-like, and counting accuracy becomes highly sensitive to ground sampling distance and flight altitude. At even larger scales, satellite imagery can encompass entire regional landscapes. However, quantitative counting from such data becomes feasible only for large, sparse entities such as individual trees in plantations, rather than dense crop organs. Overall, the choice of acquisition platform fundamentally determines the feasible counting entities, the types of annotations that can be effectively applied, and the appropriate data representation (whether bounding boxes, point annotations, density maps, or 3D volumes). This choice represents a major source of cross-domain distribution shift when deploying counting models in practice.

#### 3.4.2 Datasets

To benchmark plant counting methods, the community has released a number of public datasets based on the acquisition platforms described previously. Table 3 provides a summary of representative datasets, which vary across key dimensions including crop type, counting entity, annotation formats, image resolution, and dataset size.

Representative examples include PRL and CVPPP for Arabidopsis rosette and leaf counting [122], ACID for wheat spikelet quantification [14], URCAL for rice panicle analysis [192], and fruit or seed collections such as DateFruit [181]. In orchard fruit counting, MinneApple [24] and MangoYOLO [115] serve as widely adopted benchmarks. For grape bunch counting, WGISD [118] and wGrapeUNIPD [187] provide annotated field-based images, while Bayberry [184] offers high-res orchard images. Regarding pod, ear, and tassel counting, YOLOPOD [112] is designed for soybean pod detection, whereas MTC [21] and WSC [77] support tassel and ear counting in cluttered field conditions. More general vegetation datasets, such as CropAndWeed [171] and PhenoBench [172], provide per-pixel multi-class annotations for crops and weeds, facilitating derived counting tasks like crop or weed instance and patch counting. To cover broader spatial scales and incorporate stronger domain variation, GWHD aggregates wheat ear images from diverse field configurations and has become a standard benchmark for wheat head detection and counting [10,23]; SHC targets sorghum heads in UAV imagery [43]; and MrMT provides multi-row maize tassel images captured from elevated viewpoints [185].

Despite the growing number of available datasets, the current landscape of plant counting data remains highly fragmented across critical dimensions such as species, acquisition platforms, spatial and temporal scales, imaging modalities, and annotation protocols. Each individual benchmark is typically confined to a narrow scope, focusing on one or a few plant species, utilizing data from a single platform (e.g., a specific handheld camera, gantry system, or UAV), and adhering to a single annotation format (e.g., points, bounding boxes, instance masks, or image-level counts). This fragmentation in annotation protocols is not merely descriptive. It directly affects the supervision signal and the downstream model family. Counts at the image level are easy to scale but provide limited spatial information. Point annotations offer lightweight localization cues for dense or small targets, but do not describe object size. Bounding boxes are compatible with counting through detection for discrete organs, but become ambiguous under severe occlusion, clustering, or flexible organ morphology. Pixel level masks provide richer supervision for counting through segmentation and related phenotyping tasks, but their annotation cost and dependence on boundary definitions limit scalability. Consequently, regressors trained with point annotations, detectors trained with bounding boxes, and segmenters trained with masks may all produce count estimates, yet they learn from different spatial signals and expose different error modes. This fragmentation presents significant challenges: it hinders fair cross-dataset comparison of methodological performance, complicates the systematic evaluation of model robustness against real-world domain shifts induced by platform variability, and limits the development of models that can generalize beyond the specific species-platform combinations represented in any single, isolated benchmark.

Remark 6 **(Current limitations of plant counting)** Current plant counting benchmarks remain fragmented because datasets often deal with distinct counting entities and rely on incompatible supervision forms, such as points, boxes, masks, or density maps. As a result, cross-dataset comparison in plant counting is not directly comparable, even when models are evaluated on the same entity.

## 4 Where plant counting should go: Road toward standardization and universality

The analysis presented in Section 3 reveals a high degree of customization in existing plant counting methods, which incurs significant costs in both research and deployment. Consequently, there is a pressing need to develop standardized, general-purpose methodologies for visual plant counting. Such advancements would address the industrial requirement for standardized tools while simultaneously meeting the academic pursuit of universal counting techniques. Industrially, robust methods must deliver consistent performance across varied growth stages, spatial scales, data modalities, and crop species to facilitate scalable deployment and sustainable maintenance in practical agricultural systems. Academically, plant counting requires general-purpose visual representations that abstract away species-specific appearances and modality-dependent variations, enabling unified modeling and cumulative scientific insight. Achieving the dual goals of standardization and generalization necessitates overcoming three core challenges: cross-scale, cross-species, and cross-modal plant counting. This section outlines promising directions to address these challenges at the level of datasets, models, and evaluation protocols.

### 4.1 From niche to omni: Toward versatile plant counting datasets

Existing plant counting datasets are typically constructed for narrowly defined experimental setups, focusing on specific crop species, growth stages, platforms, or scales. While such niche datasets are valuable for benchmarking task-specific methods, their fragmented nature fundamentally limits progress toward standardized and general-purpose plant counting. To support unified counting paradigms, future datasets must be designed with explicit generalization goals, moving beyond mere increases in data volume. In particular, a versatile plant counting dataset should be organized along three core dimensions: species, scale, and modality; each of which introduce distinct design requirements (as shown in Fig. 9). Importantly, these three dimensions are not independent design axes, but mutually coupled factors.

**Fig. 9:**
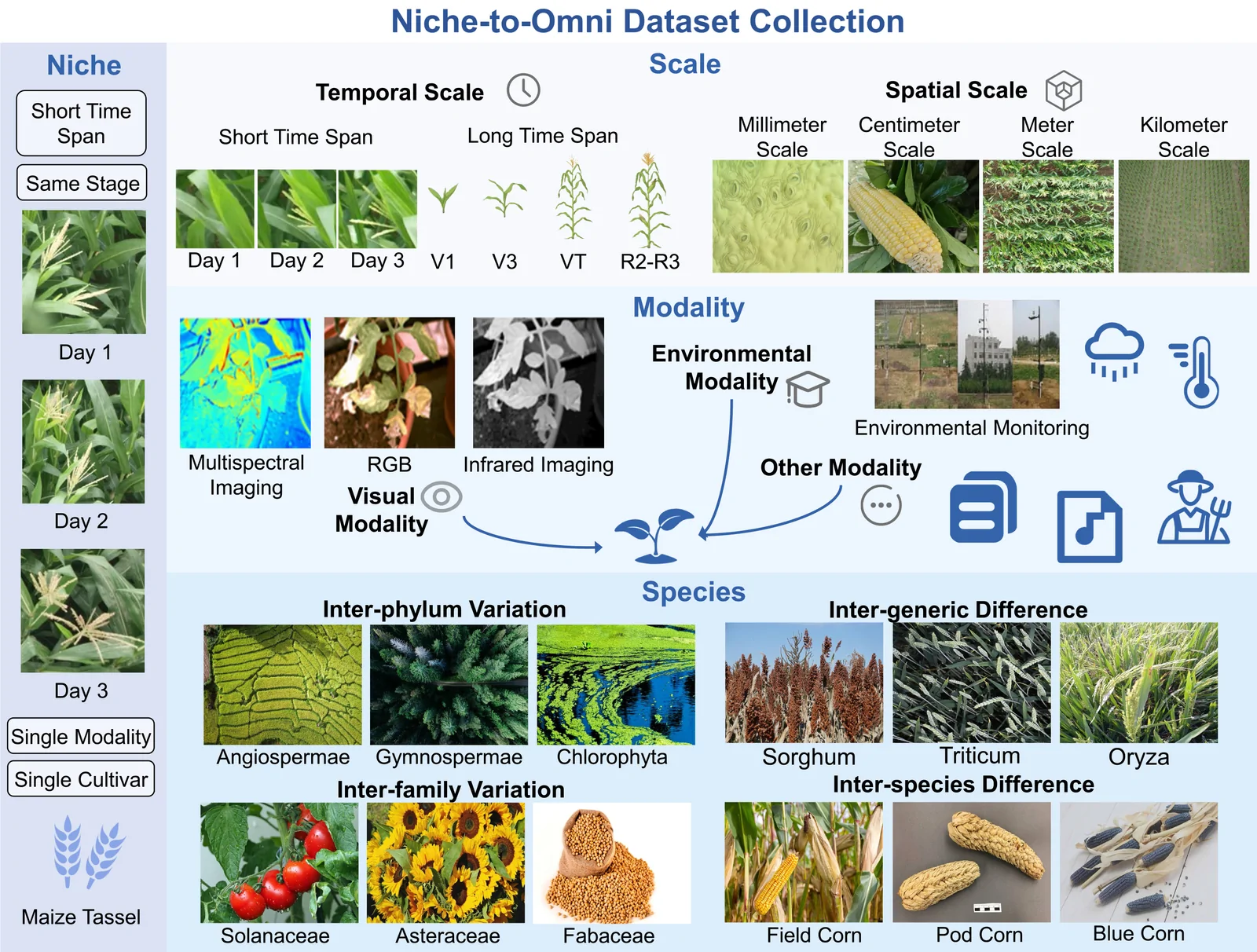
**Niche-to-omni evolution of plant counting datasets**. Current datasets (top left) are often niche, constrained by single observation timesteps, specific growth stages, or single cultivars. Future omni datasets move toward universality through three key dimensions: (a) integrating temporal scales from short-term changes to full growth cycles and spatial scales from cells to macro-level fields (b) combining visual modality data (e.g., RGB, infrared imagery, and vegetation index); and other modalities; and (c) expanding taxonomic coverage across different phyla, families, genera, and specific cultivars.

Most current plant counting datasets are built around a single species or a limited set of closely related crops, which presupposes a closed taxonomic scope. This design encourages species-dependent feature learning and hinder the development of unified counting frameworks. *A versatile dataset should instead embrace taxonomic openness*, incorporating diverse species with varying morphological traits, growth patterns, and canopy structures. Crucially, species diversity should not be treated as a mere accumulation of classes, but as a structured way to reveal shared phenotypic structures and repetitive visual patterns across taxonomic groups. By organizing data to highlight both cross-species commonalities and intra-species variations, datasets can effectively promote the learning of species-invariant counting representations.

Notably, the pursuit of taxonomic openness inherently introduces variability in both growth and observation scales, as different species possess distinct developmental rhythms and structural organization across time and space. Consequently, plant counting is fundamentally a spatiotemporal problem, where individual plants evolve continuously through developmental stages and are imaged under diverse spatial resolutions and fields of view. Most existing datasets, however, capture plants at isolated time points or fixed spatial scales, fragmenting the counting task into static subproblems. *Future versatile datasets should be constructed to embody temporal continuity and multi-scale observation, aligning data collection cycles with plant phenological processes.* This entails both short-term temporal sampling (e.g., daily or weekly) and long-term longitudinal tracking across full growth cycles, combined with spatial coverage that spans from organ-level detail to canopy- and field-level context. Such an integrated design can enable a model to learn scale-invariant representations and facilitates the study of spatio-temporal counting paradigms, including tracking-based and growth-aware counting approaches.

As both the taxonomic scope and the spatiotemporal scales increase, reliance on any single sensing modality becomes fundamentally limited. While modern plant phenotyping has widely employed heterogeneous sensors, including RGB, depth, multispectral, hyperspectral, and thermal cameras, most plant counting datasets remain confined to a single modality. This limitation restricts their applicability to real-world agricultural settings with variable sensing conditions. *A truly versatile dataset should therefore incorporate multi-modal observations with explicit cross-modal alignment*, ensuring that different sensory streams capture the same plant instances under consistent spatial and temporal conditions. Beyond visual modality, future datasets can further benefit from integrating non-visual modalities, such as environmental measurements (e.g., temperature, humidity, irradiance, and soil conditions) and structured or unstructured text (e.g., phenotyping protocols, growth-stage descriptions, or agronomic records). By associating visual observations with broader contextual signals, such datasets can enable models to ground plant appearance and structure with environmental and semantic context. Rather than treating each modality as an independent data source, such datasets should facilitate learning modality-invariant and context-aware representations by exploiting shared and complementary structural information across sensing channels. This design principle can not only improve robustness to missing or degraded modalities but also provide a basis for unified multi-modal plant counting.

The construction of a truly versatile plant counting dataset exceeds the resources and scope of any single laboratory or institution, requiring long-term collaboration at a global level. To effectively integrate the numerous fragmented datasets collected across diverse regions, climates, and experimental protocols, it is essential to establish standardized annotation guidelines, unified data formats, and open sharing platforms. Annotation design is another critical factor in the niche-to-omni transition. Different annotation formats encode different levels of biological and spatial information: image-level counts are scalable but provide weak supervision; point labels offer an efficient compromise for dense or small targets; bounding boxes support detection-based counting for discrete instances; and pixel or instance masks provide precise spatial delineation at much higher annotation cost. These choices directly shape which model families can be trained and how easily a dataset can scale across species, growth stages, and sensing platforms. Therefore, future omni-datasets should not only expand species, scales, and modalities, but also ensure that annotation cost itself remains scalable by publishing clear annotation protocols and, where possible, compatible multi-format annotations or converters between points, boxes, and masks. From our perspective, point annotations provide a particularly economical and compatible basis for large-scale plant counting datasets, because they can support regression-based counting directly and can also serve as prompts for generating bounding boxes or masks with foundation segmentation models such as the Segment Anything Model (SAM) [194]. Beyond standardized collection and sharing, a collaborative plant counting resource would benefit from a queryable database schema rather than a simple list of datasets. Similar design principles are visible in mature data management platforms. For example, TCIA and PhysioNet organize multiple datasets with standardized metadata, versioned access, and explicit provenance, enabling users to retrieve resources by task-relevant criteria rather than manual searching [195,196]. These examples suggest that a collaborative plant counting database should explicitly encode species, organ type, growth stage, spatial scale, platform, sensor, annotation format, and provenance, so that researchers can rapidly identify all datasets matching a specific review or task requirement. Encouragingly, recent community efforts are beginning to align with this vision. For example, the ImAg4Wheat initiative [34] aggregates imagery from over 30 global sites, thereby exposing models to wide genotypic, environmental, and site-level variation and making cross-site robustness more measurable. Datasets such as TPC-268 [197] and PAC-105 [33] further explore taxonomy-aware and plant-agnostic counting by expanding the range of species and weakening the dependence on a single crop-organ definition. These design choices suggest that broader transferability requires not only more samples, but also deliberately structured variation across species, environments, platforms, and counting entities. Nevertheless, current resources remain partial steps toward omni-datasets, because their coverage of sensing modalities, growth stages, and biological scales is still limited. They therefore provide valuable prototypes rather than complete solutions for future omni-scale, omni-species, and omni-modal dataset construction.

Remark 7 **(In pursuit of niche-to-omni plant counting datasets)** Future plant counting datasets should embrace taxonomic openness, temporal continuity with multi-scale observation, and explicit cross-modal alignment. Together, these properties define whether a dataset can support generalizable plant counting rather than isolated counting solutions for single species, scales, or modalities.

### 4.2 From customization to unification: Toward unified plant counting foundation models

A unified model architecture, built upon versatile datasets, is essential for advancing the standardization and universality of plant counting. As outlined in Section 3.1, however, current methods rely heavily on iterative customization conditioned on species, organs, and growth stages, resulting in models that are inherently task-specific and species-dependent. Transitioning from customized solutions to a unified counting model, therefore, demands a fundamental reformulation of the problem per se. As conceptualized in Fig. 10, the core question should shift from *how to count a particular plant* to *how to learn to count a plant*, independent of species identity, imaging scale, and sensor modality. This reformulation moves the central challenge from task-specific model design to the acquisition of a generalizable visual counting capability.

**Fig. 10:**
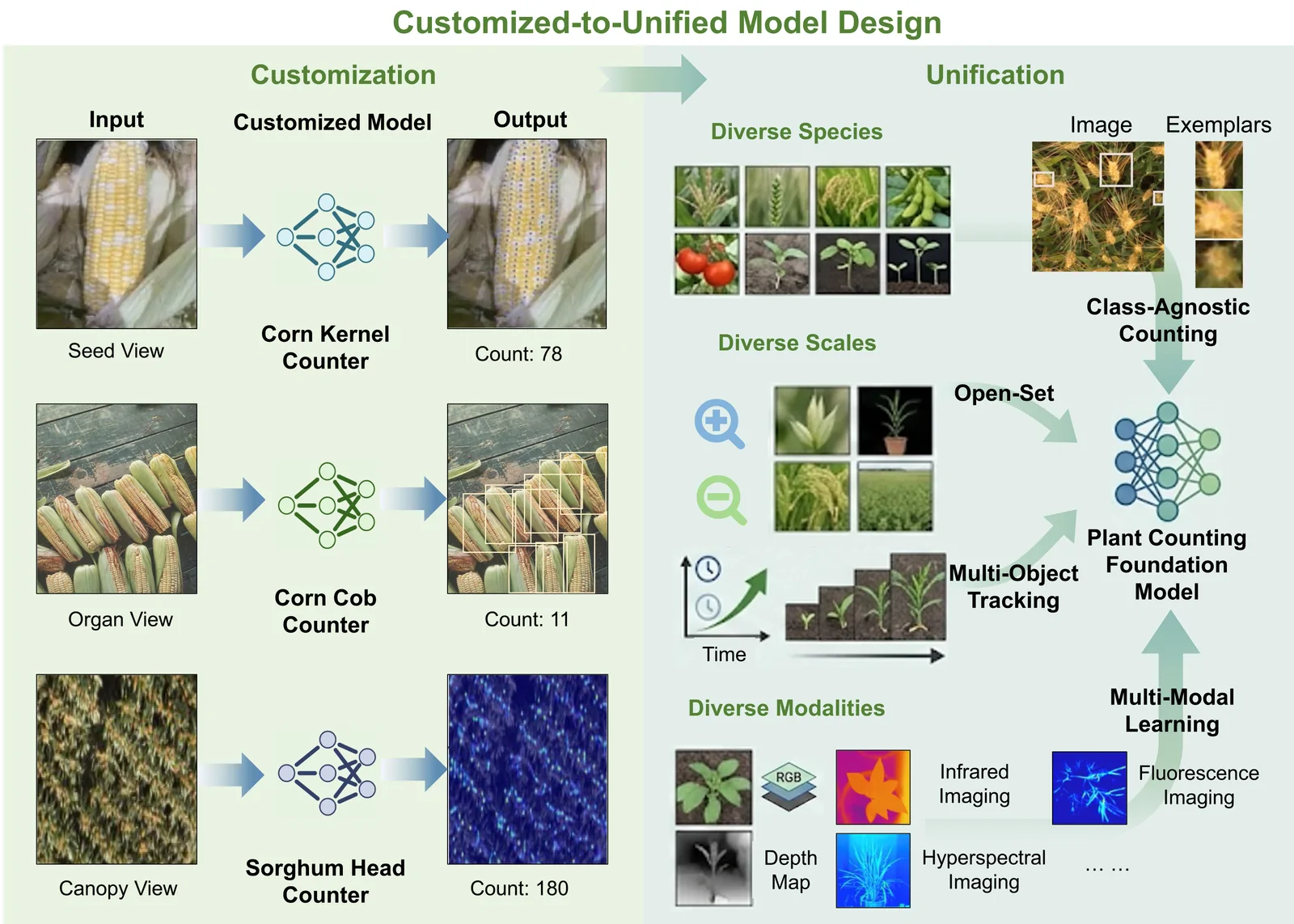
**Customized-to-unified evolution of plant counting models**. Conventional approaches (left) are typically tailored to specific species and observation scales (e.g., seed, organ, or canopy level), necessitating distinct models and annotations for each task. The future unified paradigm (right) reformulates plant counting as an instance-centric perception problem. By integrating class-agnostic counting, scale-invariant modeling (e.g., via MOT or open-set learning), and modality-invariant representation learning, a unified plant counting foundation model can generalize across species, spatial scales, and heterogeneous sensing modalities.

A promising pathway toward this goal is offered by class-agnostic counting (CAC) [[30], [31], [32]], which have emerged in general computer vision to count objects from arbitrary categories. CAC reformulates counting as a problem of exemplar matching: given a query image and a few exemplars, the model extracts feature representations and computes a similarity map to localize all instances matching the exemplars. Early implementations used convolutional neural networks for feature extraction and inner-product-based similarity computation [30]. Subsequent work extended matching to multiple scales [198,199] or introduced explicit similarity guidance maps [31,32,200]. For plant counting, cross-species generalization should be understood operationally: a model should maintain reliable counting performance when transferred to unseen species or unseen species–organ combinations, provided that the target entity is specified by exemplars or other task conditions rather than by fixed class labels. This requirement shifts the problem from closed-set category recognition to conditional counting under morphological variation. Inspired by this paradigm, recent work has introduced plant-agnostic counting (PAC) [33], which decouples the counting mechanism from species-specific definitions and proposes TasselNetV4 to achieve cross-species counting. This shift is particularly meaningful for plant counting, as it allows counting to be formulated based on *exemplar-driven semantic conditions rather than predefined taxonomic labels*, thereby enabling a unified framework for counting plants with diverse morphologies and growth stages. However, current PAC-style methods mainly address semantic transfer across categories; broader universality also requires robustness to changes in observation scale, sensor modality, and temporal stage.

Scale variation poses a second major challenge for standardization. For plant counting, cross-scale generalization should be operationally defined as the ability to maintain reliable counts when the same biological target is observed at different spatial resolutions, fields of view, or temporal sampling intervals, without requiring a complete redesign of the counting objective or annotation protocol. An ideal system should possess *real-time perceptual adaptability and dynamic environmental awareness* to tackle cross-scale counting. This system should be explicitly designed to learn scale-invariant representations, ensuring that plant instances observed at different spatial or temporal resolutions are mapped to a consistent feature space. Recent progress in MOT for plants [201,202] shows that counting can be achieved naturally through temporal association and trajectory accumulation, which improves transferability from single-frame recognition to long-term observation. This design is useful for cross-scale and temporal generalization because the model can accumulate object evidence across views or time rather than relying on one static image. Beyond counting-specific MOT pipelines, foundation-model segmentation in plant time-series [203] also demonstrates that few-shot prompting and temporal mask propagation can maintain stable instance-level visual consistency across strong growth-driven deformation, providing transferable cues for temporally consistent counting. However, these approaches still depend on reliable association, stable imaging intervals, and sufficiently consistent target semantics; they may fail when occlusion is severe, growth changes the identity of countable entities, or the observation scale shifts beyond the training regime. Therefore, *plant counting models must be grounded in theory to operate robustly under open-set conditions across spatial scales*, where the observation scale may not be fully known during training. A promising direction is dual-level scale modeling, which combines semantic-level representations that capture organ-individual-canopy hierarchies with visual-level mechanisms that use scale-aware priors to suppress irrelevant scales and amplify target-scale responses. Such a design can bridge the gap between closed-set and open-set scenarios and advance plant counting from scale-dependent heuristics toward a principled, general-purpose paradigm.

Finally, cross-modal variation challenges the feasibility of a unified representation, as plant appearances differ drastically across sensing modalities. The cross-modal objective is therefore to *build models that learn modality-invariant representations*, drawing on multimodal techniques from computer vision [204,205]. These models should be trained to extract counting-relevant information while discarding modality-specific appearance variations. This direction is also supported by recent agricultural vision foundation models. For example, SPROUT [206] demonstrates that diffusion-based pre-training on large-scale unlabeled agricultural imagery can substantially reduce domain gaps between generic web-pretrained vision foundation models and real agricultural scenarios, while improving performance across diverse downstream tasks at lower pre-training cost. Complementary evidence comes from Refs. [207,208], which leverages Depth Anything to generate depth-informed pseudo labels and reaches near fully supervised crop segmentation performance with much lower manual annotation cost, indicating that agriculture-adapted foundation priors can be translated into practical phenotyping pipelines. These findings provide practical evidence that foundation-model pretraining tailored to agricultural data is a key step toward unified and transferable plant counting systems. Initial explorations in multimodal counting [209,210] and plant phenotyping [211] leverage heterogeneous inputs, such as RGB, depth, and even textual descriptions, to improve robustness. Further exploration into cross-modal alignment and fusion mechanisms is critical to achieve strong modality-invariant feature learning.

Remark 8 **(Toward customized-to-unified plant counting models)** Plant counting should move beyond customized counters built for specific species or organs toward a unified counting mechanism that remains valid across species, scales, and sensing modalities. In this sense, class-agnostic matching, scale-aware modeling, and modality alignment matter because they help preserve the same counting rule under changing biological counting entities and imaging conditions.

### 4.3 From local to global: Toward systematic assessment of plant counting models

Despite steady progress in plant counting models, prevailing evaluation protocols remain largely local and closed-world. Most studies report performance under controlled environments or narrowly constrained field settings (see Fig. 11), typically involving limited cultivars, a single acquisition platform, and homogeneous environmental conditions. While such localized evaluations offer convenience and reproducibility, they carry inherent limitations. First, they assume that test data are drawn from the same distribution as the training data, which fails to reveal the sensitivity of a model to domain shifts induced by environmental variation, phenological change, and differences in sensing hardware. Second, evaluation metrics are often limited to aggregate error measures such as mean absolute error (MAE) or root-mean-square error (RMSE), which can obscure systematic failure modes, including sensitivity to scale variation, occlusion, or biases specific to certain growth stages. Consequently, even models that achieve strong benchmark performance may offer limited insight into their real-world deployment reliability.

**Fig. 11:**
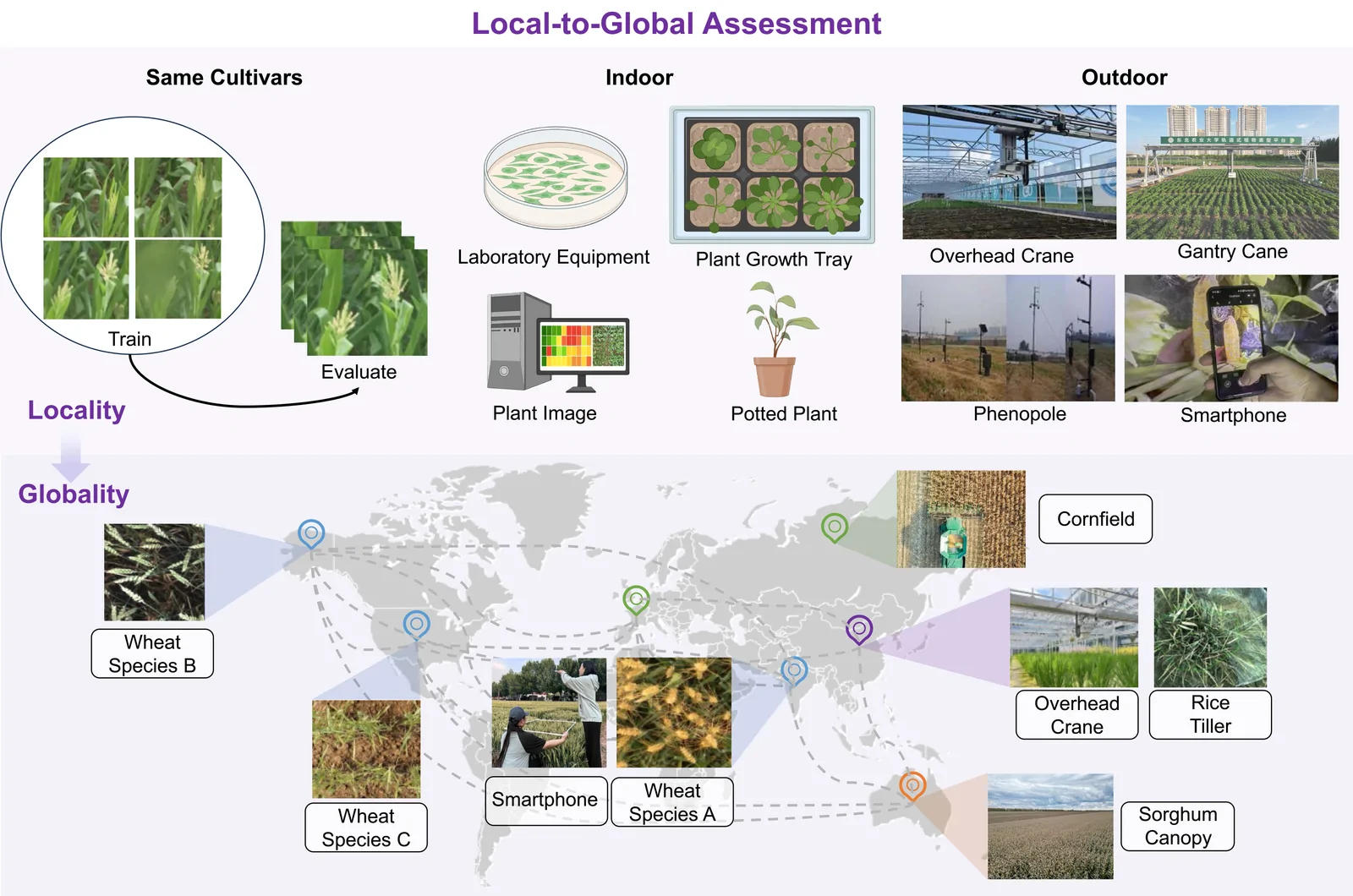
**Local-to-global evolution of plant counting assessments**. Current plant counting models are often developed and evaluated under localized conditions, such as controlled indoor environments (laboratories and greenhouses) or limited field setups with uniform cultivars and fixed acquisition platforms. Future assessment frameworks should instead validate models across diverse environments, sensing platforms, geographic regions, and genetic backgrounds to broadly assess robustness and generalizability.

In practical agricultural scenarios, however, plant counting models must operate under open and dynamically varying conditions that span diverse environments, management practices, and genetic backgrounds. As noted in recent plant-science perspectives [35], performance under controlled conditions is an insufficient predictor of agronomic utility. Bridging this gap requires a paradigm shift not only at the dataset and model levels, but also in model assessment—from locality to globality. Rather than optimizing performance on a single, static benchmark, global assessment seeks to characterize model robustness, generalization capacity, and failure patterns across heterogeneous, geographically distributed scenarios, mirroring the philosophy of multi-environment trials in crop breeding.

A systematic global assessment framework should integrate three core components. First, *evaluation should span multi-site and multi-season field data* to explicitly quantify generalization across environmental and temporal variations. Second, *models should be tested across heterogeneous sensing platforms and observation scales* to diagnose sensitivity to variations in resolution, viewpoint, and acquisition modality. Third, assessment should advance beyond number-based accuracy or error metrics toward diagnostic evaluation, which stratifies errors by factors such as growth stage, planting density, and environmental context. Such a protocol transforms evaluation from a mere ranking exercise into a diagnostic tool for understanding model behavior and informing practical deployment decisions.

Admittedly, a fully fair and fully global assessment protocol is often difficult to realize in plant counting, because instance semantics and counting objectives may change across platforms, scales, and biological targets. Approximating this ideal requires multi-site, multi-season, and cross-platform evaluation, together with condition-aware error analysis across growth stages, density levels, and environmental contexts. Nevertheless, existing studies are already moving toward this goal. In plant phenotyping, analyses explicitly characterize temporal and cross-site domain shifts when deploying image-based models in the field [22]. In a broader agricultural vision, benchmarks such as Fields of the World explicitly structure training and testing across countries to study zero-shot transfer and fine-tuning under agro-ecological diversity [212]. In parallel, recent work on global plant diversity modeling [213] has integrated over 830 regional plant inventories worldwide and evaluated models across diverse climate, environmental, and biogeographic conditions. Although these efforts do not yet constitute fully standardized global assessment frameworks, they indicate a valuable direction from localized evaluation toward systematic, deployment-oriented plant counting assessment. At present, plant counting has not yet converged on a standardized multi-dimensional robustness metric comparable to MAE or RMSE. A practical near-term solution is to report domain-conditioned errors and transfer gaps. Specifically, benchmark reports could include in-domain error, leave-one-site-out error, leave-one-season-out error, leave-one-platform-out error, worst-domain error, and relative performance degradation between in-domain and out-of-domain settings. For counting tasks, these dimensions can be implemented as stratified MAE/RMSE values, such as MAE_*site*_, MAE_*season*_, and MAE_*platform*_. Such a robustness metrics would not replace aggregate counting accuracy, but would reveal whether a model fails because of site shift, phenological shift, platform shift, scale mismatch, or target-definition drift.

Remark 9 **(Rethinking local-to-global plant counting assessment protocols)** Local plant counting benchmarks mainly test in-domain fit, whereas global assessment tests whether the same counting rule holds across sites, seasons, cultivars, and sensing platforms. For plant counting, this shift matters because failure often appears as systematic counting errors, such as under-estimation in dense scenes, over-estimation under occlusion, or counting drift across growth stages and scales.

## 5 Conclusion

Plant counting is a fundamental task in plant phenotyping, forming an essential link between raw visual observations and downstream biological and agronomic analyses. In this review, we have systematically revisited the evolution of plant counting methodologies by organizing the literature along three dimensions: the historical origins of the problem, the current state of solutions, and the critical directions for future advancement.

Historically, plant counting methods have evolved from early rule-based and handcrafted feature extraction to modern deep learning paradigms, driven largely by advances in computer vision. These developments have delivered substantial gains in accuracy within controlled environments and for specific crop scenarios. However, as our analysis reveals, much of this progress remains constrained by customized problem formulations, narrow data distributions, and localized evaluation protocols.

An examination of the current state of the art reveals several structural limitations that hinder the practical scalability and scientific generalization of plant counting. First, most existing methods rely heavily on task-specific customization across species, observation scales, and imaging platforms, leading to fragmented solutions rather than transferable knowledge. Second, evaluation practices are predominantly local, often constrained to single datasets or controlled environments, providing limited insight into real-world robustness under heterogeneous agricultural conditions. Third, the lack of standardized datasets, benchmarks, and evaluation protocols makes it difficult to fairly compare methods or to assess genuine progress at the field level.

Looking forward, we argue that the future of plant counting lies in a paradigm shift from customized and localized solutions toward unified, standardized, and generalizable counting frameworks. Central to this transition is the construction of versatile plant counting datasets that span scales, modalities, species, and environmental conditions, enabling the development of counting foundation models with stronger generalization ability. In parallel, systematic and global evaluation protocols are required to move beyond isolated performance gains and toward reliable deployment in real agricultural production. Moreover, integrating domain knowledge from plant science with data-driven modeling, knowledge-guided architectures, and adaptive learning strategies—will be essential for bridging the gap between visual counting outputs and biological interpretation.

Ultimately, advancing plant counting is not merely a technical challenge but a foundational step toward standardized visual plant phenotyping. By rethinking problem formulation, data construction, model design, and evaluation strategies in a unified manner, plant counting has the potential to evolve from a collection of task-specific solutions into a core component of AI-driven plant phenomics systems. We hope this review can serve as a reference point for both the computer vision and plant science communities, fostering closer interdisciplinary collaboration and accelerating the development of robust, scalable, and biologically meaningful plant counting technologies.

## Author contributions

H. Lu – Conceptualization, Analysis/Synthesis, Writing – Original Draft, Writing – Review & Editing, Supervision, Funding Acquisition.

W. Zhang – Literature Search & Curation, Visualization, Analysis/Synthesis, Writing – Original Draft.

M. Yang – Literature Search & Curation, Visualization, Analysis/Synthesis, Writing – Original Draft.

## Data availability

N/A.

## Funding

This work is supported by the 10.13039/501100001809National Natural Science Foundation of China under Grant No. 62576146.

## Declaration of competing interest

The authors declare that they have no known competing financial interests or personal relationships that could have appeared to influence the work reported in this paper.

The author is an Editorial Board Member/Editor-in-Chief/Associate Editor/Guest Editor for this journal and was not involved in the editorial review or the decision to publish this article.
